# Heteroatom Substitution at Amide Nitrogen—Resonance Reduction and HERON Reactions of Anomeric Amides

**DOI:** 10.3390/molecules23112834

**Published:** 2018-10-31

**Authors:** Stephen A. Glover, Adam A. Rosser

**Affiliations:** Department of Chemistry, School of Science and Technology, University of New England, Armidale, NSW 2351, Australia; arosser3@une.edu.au

**Keywords:** amide resonance, anomeric effect, HERON reaction, pyramidal amides, physical organic chemistry, reaction mechanism

## Abstract

This review describes how resonance in amides is greatly affected upon substitution at nitrogen by two electronegative atoms. Nitrogen becomes strongly pyramidal and resonance stabilisation, evaluated computationally, can be reduced to as little as 50% that of *N*,*N*-dimethylacetamide. However, this occurs without significant twisting about the amide bond, which is borne out both experimentally and theoretically. In certain configurations, reduced resonance and pronounced anomeric effects between heteroatom substituents are instrumental in driving the HERON (Heteroatom Rearrangement On Nitrogen) reaction, in which the more electronegative atom migrates from nitrogen to the carbonyl carbon in concert with heterolysis of the amide bond, to generate acyl derivatives and heteroatom-substituted nitrenes. In other cases the anomeric effect facilitates S_N_1 and S_N_2 reactivity at the amide nitrogen.

## 1. Introduction

**Figure 1:**
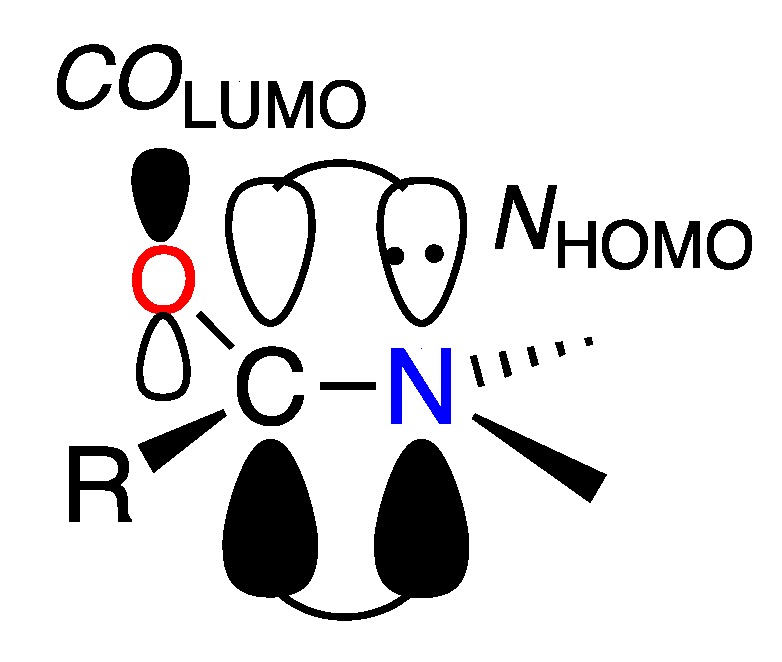
The interaction between the nitrogen highest occupied molecular orbital (HOMO) and the carbonyl lowest unoccupied molecular orbital (LUMO) in amides.

**Figure 2:**
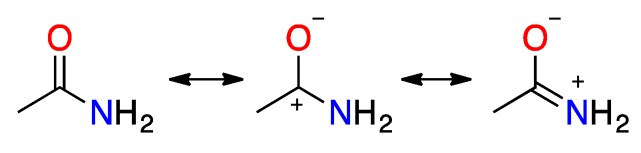
Resonance hybrid contributions in amides, showing that the majority of charge transfer occurs between nitrogen and carbon.

Amides are prevalent in a range of molecules such as peptides, proteins, lactams, and many synthetic polymers [1]. Generically, they are composed of both a carbonyl and an amino functional group, joined by a single bond between the carbon and nitrogen. The contemporary understanding of the resonance interaction between the nitrogen and the carbonyl in amides is that of an interaction between the lowest unoccupied molecular orbital (LUMO) of the carbonyl, π*_C=O_, and the highest occupied molecular orbital (HOMO) of the amide nitrogen (N2p_z_) (Figure 1). This molecular orbital model highlights the small contribution of the carbonyl oxygen to the LUMO, which indicates that limited charge transfer to oxygen occurs, in line with the resonance model presented in Figure 2, which signifies that charge at oxygen is similar to that in polarized ketones or aldehydes and nitrogen lone pair density is transferred to electron deficient carbon rather than to oxygen. The major factor in the geometry of amides, and the restricted rotation about the amide bond, is the strong π-overlap between the nitrogen lone pair and the C2p_z_ component of the LUMO, which dominates the π*_C=O_ orbital, on account of the polarisation in the π_C=O_ [2,3].


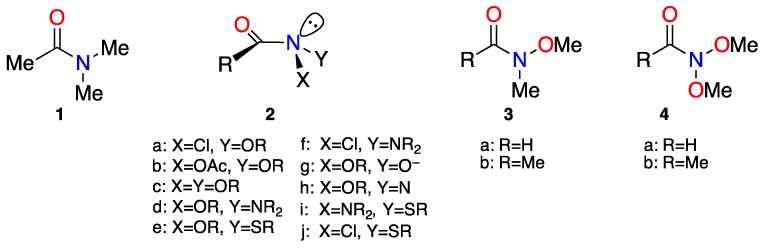


Amide resonance can be diminished by limiting the overlap between the nitrogen lone pair, n_N_, and π*_C=O_ orbitals. In most instances, this can occur by twisting the amino group about the N–C(O) bond and/or pyramidalising the nitrogen, which amounts to introducing “s” character into the N2p_z_ orbital.

**Figure 3:**
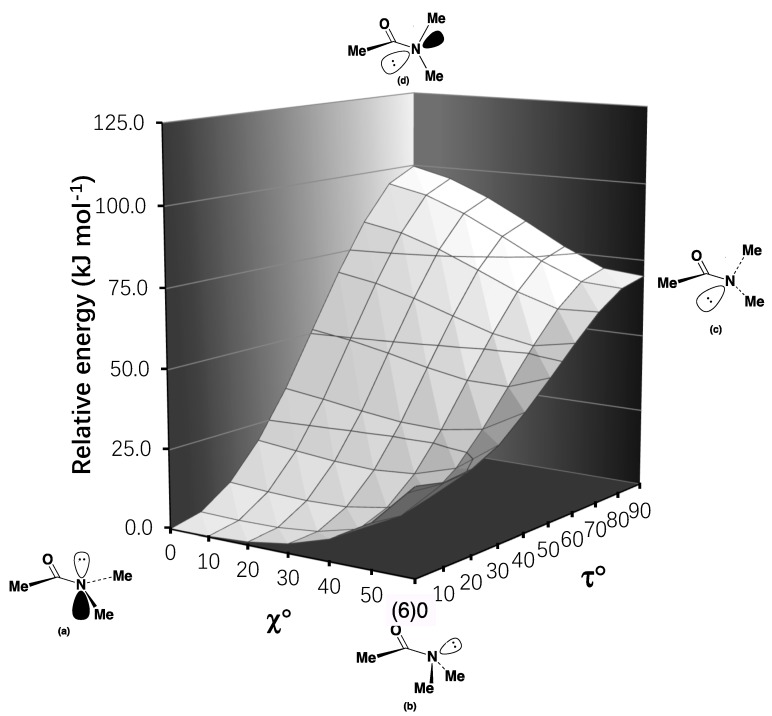
A B3LYP/6-31G(d) generated energy surface for the deformation of the amide moiety in *N*,*N*-dimethylacetamide 1 [4]. χ and τ are Winkler–Dunitz parameters for pyramidalisation and twist, respectively [5,6]. (**a**) The ground state planar structure for *N*,*N*-dimethylacetamide; (**b**) untwisted system with sp^3^ hybridised nitrogen; (**c**) 90° rotation about N–C(O), whilst maintaining sp^3^ hybridisation at nitrogen; and (**d**) the fully twisted moiety, 90° N–C(O) rotation and sp^2^ hybridised nitrogen.

Computational modelling of N–C(O) rotation and nitrogen pyramidalisation in *N*,*N*-dimethylacetamide **1**, at the B3LYP/6-31G(d) level, illustrates the energetic changes (Figure 3) [4]. Distortion of the amide linkage can be quantified by Winkler–Dunitz parameters χ and τ, where χ = 60° for a fully pyramidal nitrogen, and τ = 90° for a completely twisted amide where the lone pair orbital on nitrogen is orthogonal to the C2p_z_ orbital [5,6]. Deformation from the non-twisted, sp^2^ planar ground state (Figure 3a) through rehybridisation of nitrogen to sp^3^ (Figure 3b) is accompanied by an increase in energy (~27 kJ mol^−1^), but the majority of amide resonance remains intact. A much larger increase in energy (~100 kJ mol^−1^) results from twisting the N–C(O) bond through 90° whilst maintaining sp^2^ planarity at nitrogen (Figure 3d), and as the nitrogen is allowed to relax to sp^3^ hybridisation (Figure 3c), a fully twisted amide devoid of resonance is obtained. The loss of ~31 kJ mol^−1^ in this final step is indicative of the concomitant twisting and pyramidalisation observed in a variety of twisted amides.

Typically, amides exhibit restricted rotation about the N–C(O) bond, in the order of 67–84 kJ mol^−1^, along a sigmoid pathway with little change in energy upon moderate pyramidalisation (χ = 0–40°) and minor twisting (τ = 0–20°). Clearly, rotation without pyramidalisation is energetically unfavourable and many examples of twisted amides are testament to this. The shift to sp^3^ hybridisation at the amidic nitrogen is clearly demonstrated when twisting of the N–C(O) bond is geometrically enforced by tricyclic and bicyclic bridged lactams [7,8,9,10,11,12,13]. Kirby’s “most twisted amide” 1-aza-2-adamantanone, synthesised in 1998 [10,12], and Tani and Stoltz’s 2-quinuclidone, synthesised in 2006 [14], exemplify the fully twisted amide, geometrically and chemically. Intramolecular steric hindrance is another source of non-planar twisted amides [15,16], as exemplified by the thioglycolurils [17,18] and other systems. Ring strain in nontwisted amides, such as 1-acylaziridines [19,20] and *N*-acyl-7-azabicyclo[2.2.1]heptanes [21,22,23,24] can result in pyramidality at the amide nitrogen, despite retaining a noticeable n_N_–π*_C=O_ interaction.

The structural changes accompanying the loss of amide resonance include the lengthening of the N–C(O) bond and minor shortening of the (N)C=O bond. Comparing the fully twisted 1-aza-2-adamantanone to an analogous unstrained tertiary δ-lactam, the N–C(O) bond shortens from 1.475 Å to 1.352 Å, and the (N)C=O bond lengthens from 1.196 Å to 1.233 Å [12,25]. Spectroscopically and chemically, the amide carbonyl trends towards ketonic behaviour at large twist angles. In tricyclic 1-aza-2-adamantanone, the carbonyl carbon ^13^C NMR resonance is at 200.0 ppm and the IR carbonyl vibrational frequency (1732 cm^−1^) is significantly higher than regular amides (1680 cm^−1^) [10,12].

## 2. Properties of Anomeric Amides

### 2.1. Structural Properties

Another way in which amides may be dispossessed of their planarity and resonance is through bisheteroatom-substitution by electronegative heteroatoms at the amide nitrogen in **2**. We named this class ‘anomeric amides’ on account of the pronounced anomeric effects that can and do occur between the heteroatoms [26,27,28,29,30,31,32,33,34,35]. However, the physical, theoretical, and chemical properties of various congeners differ from those of conventional primary, secondary, and tertiary alkylamides.

**Figure 4:**
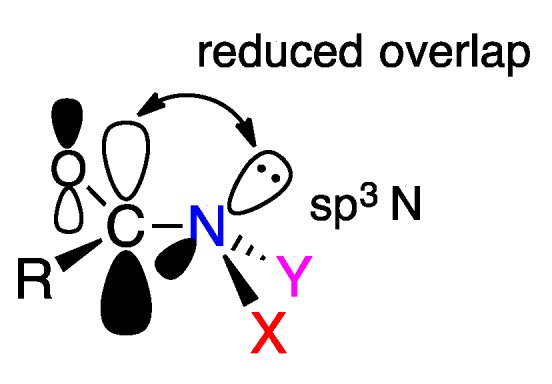
Reduced n_N_–π*_C=O_ overlap due to pyramidalisation.

Electronegative atoms demand an electron density redistribution which is facilitated by a shift in the hybridisation at nitrogen towards sp^3^, in accordance with Bent’s rule [36,37]. Reduced n_N_–π*_C=O_ overlap due to pyramidalisation (Figure 4) together with the increased ‘2s’ character of the lone pair on nitrogen results in structural, electronic, spectroscopic, and chemical differences in comparison to traditional amides. Their unique properties of have been reviewed in recent years [31,35].

Much of our interest into, and indeed the discovery of, anomeric amide chemistry emanates from our investigations of the biological activity, structure, and reactivity of *N*-acyloxy-*N*-alkoxyamides (NAA’s) **2b** [31,38,39,40,41,42,43,44,45,46,47,48,49] a class of anomeric amides. Readily synthesised by treatment of *N*-alkoxy-*N*-chloroamides **2a**, themselves a form of anomeric amide, with silver or sodium carboxylate salts in anhydrous solvents [45,46,48,49], NAA’s are direct-acting mutagens which react with nucleophilic centres in DNA [31,39,40,41,42,43,44,45,46,49,50]. Additionally, they react with a variety of nucleophiles to produce other congeners, including, reactive anomeric amides in the form of *N*-alkoxy-*N*-aminoamides **2d** [44,47,51] and *N*-alkoxy-*N*-thioalkylamides **2e** [52]. We have also encountered several anomeric reactive intermediates through reactions of *N*-acyloxy-*N*-alkoxyamides: reaction of **2b** with base generates *N*-alkoxy-*N*-hydroxamate anions **2g** [45] and reaction with azide ultimately generates 1-acyl-1-alkoxydiazenes, which are aminonitrenes **2h** [53,54]. We have generated *N*,*N*-dialkoxyamides **2c** in related studies by solvolysis of *N*-alkoxy-*N*-chloroamides **2a** in aqueous alcohols and through the reaction of hydroxamic esters with hypervalent iodine reagents in appropriate alcohols [30,55,56]. Other anomeric amides of theoretical interest to us are *N*-amino-*N*-chloroamides **2f** [34], as well as *N*-amino-*N*-thioalkylamides **2i** and *N*-chloro-*N*-thioalkyamides **2j**.

The impact of heteroatom substitution at amide nitrogen is clearly demonstrated by comparing *N*,*N*-dimethylacetamide **1** to *N*-methoxy-*N*-methylacetamide **3b** and *N*,*N*-dimethoxyacetamide **4b**. In 1996, we reported on the B3LYP/6-31G(d) theoretical properties of the corresponding formamides **3a** and **4a** [27]. The substitution of hydrogen by methoxyl in **3a** led to an increase in N–C(O) bond length from 1.362 Å to 1.380 Å and a second substitution at nitrogen in **4a** resulted in a similar increase to 1.396 Å; the carbonyl bond contracted marginally by 0.006 Å. Nitrogen in *N*-methoxy-*N*-methylformamide and *N*,*N*-dimethoxyformamide becomes distinctly pyramidal with average angles at nitrogen of ~114°. With similar degrees of pyramidality (χ_N_), the increased N–C(O) bond length in *N*,*N*-dimethoxyformamide could not solely be attributed to deformation at nitrogen. Energetic lowering of the lone pair electrons is also responsible for reduced overlap with the adjacent C2p_z_ orbital. This is dramatically exemplified by the rotational barriers for *N*,*N*-dimethylformamide, *N*-methoxy-*N*-methylformamide, and *N*,*N*-dimethoxyformamide, which were computed to be of the order of 75, 67, and 29 kJ mol^−1^, respectively [27].

**Figure 5:**
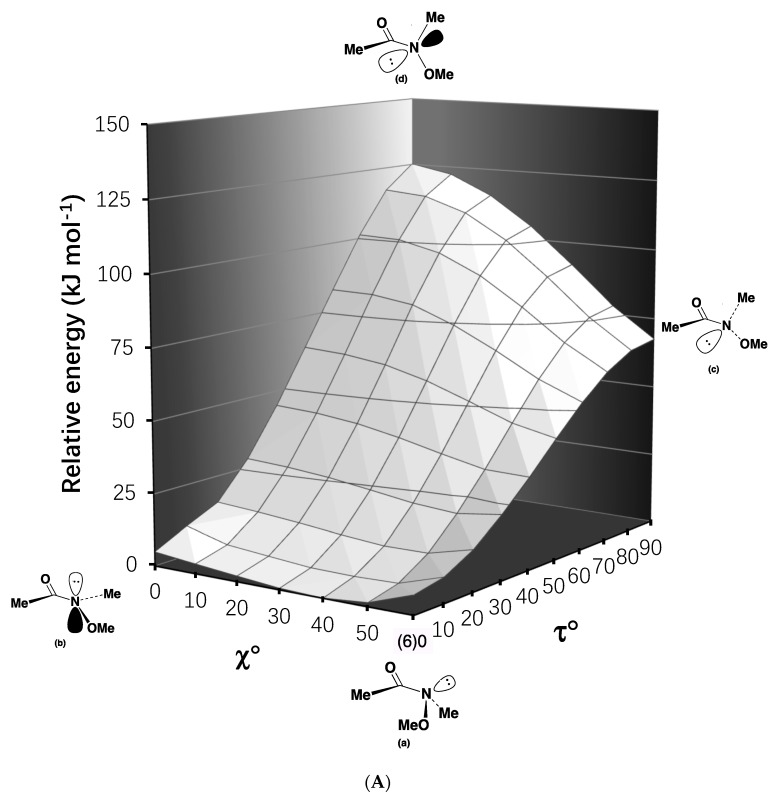

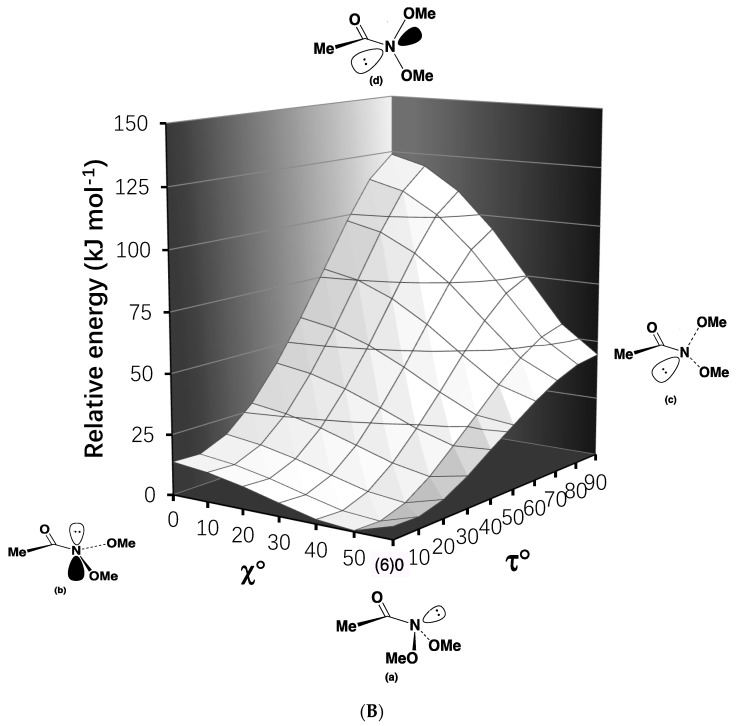
B3LYP/6-31G(d) energy surfaces for deformation of (**A**) *N*-methoxy-*N*-methylacetamide **3b** and (**B**) *N*,*N*-dimethoxyacetamide **4b**. (**a**) Untwisted system with sp^3^ hybridised nitrogen; (**b**) planar untwisted structure; (**c**) 90° rotated structure with sp^3^ hybridisation at nitrogen; and (**d**) the fully twisted moiety with sp^2^ hybridised nitrogen.

Deformation energy surfaces for *N*-methoxy-*N*-methylacetamide **3b** and *N*,*N*-dimethoxyacetamide **4b**, for comparison with that of *N*,*N*-dimethylacetamide **1**, are depicted in Figure 5. In contrast to *N*,*N*-dimethylacetamide (Figure 3), the lowest energy forms clearly deviate from planarity at nitrogen with χ_0_ in the region of 40° and 50°, respectively. In both structures, the highest point corresponding to χ = 0°, τ = 90° is metastable, and the planar fully twisted, and therefore nonconjugated forms, relax to fully pyramidal conformations (τ = 90°, χ = 60°), the energy of which reflects the B3LYP/6-31G(d) barriers to amide isomerisation in each case, which are approximately 67 and 44 kJ mol^−1^, respectively. While the energy lowering for *N*-methoxyacetamide is modest, the attachment of two electronegative oxygens to the amide nitrogen radically lowers the isomerisation barrier by some 29 to 33 kJ mol^−1^. Inversion barriers at nitrogen are low on account of the gain in resonance stabilisation in the planar form, though the barrier is higher for *N*,*N*-dimethoxyacetamide where the resonance capability would be less and a six π-electron repulsive effect would operate; planarisation in the hydroxamic ester is less costly than in *N*,*N*-dimethoxyacetamide (~6.3 vs. 14.6 kJ mol^−1^, respectively).

In a recent publication, we outlined two concurring isodesmic methods for estimating the amidicity of amides and lactams [4]. Carbonyl substitution, nitrogen atom replacement (COSNAR), developed by Greenberg evaluates the energy stabilisation when the target amide is generated from corresponding ketone and amine according to isodesmic Equation (1) [57,58,59]. Steric or substituent effects are conserved throughout the reaction so steric corrections are not required.
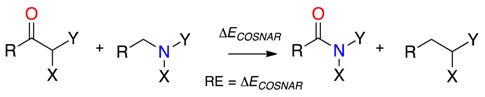
(1)

In the second approach, the transamidation method (TA), the energy change is determined when *N*,*N*-dimethylacetamide **1** transfers the carbonyl oxygen to a target amine according to Equation (2). The limiting energy increase for formation of fully twisted, unstrained 1-aza-2-adamantanone by the corresponding reaction with 1-azaadamantane, constitutes complete loss of resonance stabilisation (Δ*E*_TA_ = 76.0 kJ mol^−1^). However, where heteroatom substituents are present at nitrogen, Δ*E*_TA_ must be corrected for any additional inductive destabilisation of the carbonyl (Δ*E*_ind_) in the absence of resonance, which we obtain isodemically from reactions such as Equation (3) [33].

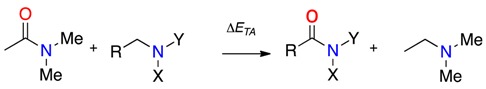
(2)

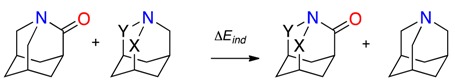
(3)

By the TA method, the residual resonance, RE_TA_, is given by Equation (4).

RE_TA_ = −76.0 kJ mol^−1^ + (Δ*E*_TA_ − Δ*E*_ind_) (4)

In both the TA and COSNAR approaches, zero point energies largely cancel and meaningful results are obtained without the need for frequency calculations [58,60]. RE by both methods, the negative of the traditional representation of resonance stabilisation energy, should correlate [4,30,33,34,61,62] and RE as a percentage of −76.0 kJ mol^−1^ (or −77.5 kJ mol^−1^ in the case of COSNAR) yields the amidicity relative to *N*,*N*-dimethylacetamide **1** (by definition 100%).

The resonance energy and amidicity of *N*-methoxy-*N*-methylacetamide **3b** has been determined with COSNAR (−62.1 kJ mol^−1^, 80% amidicity) and TA (−61.25 kJ mol^−1^, 81% amidicity) and accords nicely with the lowest rotational barrier from Figure 5a of 67.5 kJ mol^−1^. In contrast, for the bisoxyl-substituted acetamide, the RE**_COSNAR_** and RE**_TA_** were determined at −35.9 kJ mol^−1^, respectively just 47% or 46% that of *N*,*N*-dimethylacetamide [33]. From Figure 5b the rotational barrier was 44.4 kJ mol^−1^ and therefore most of the barrier can be accounted for by loss of resonance.


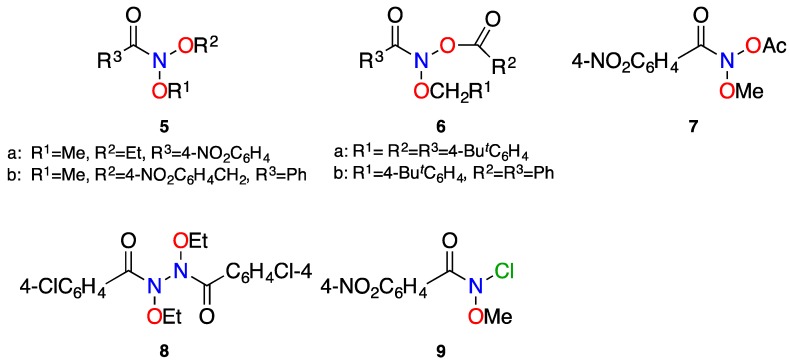


**Figure 6:**
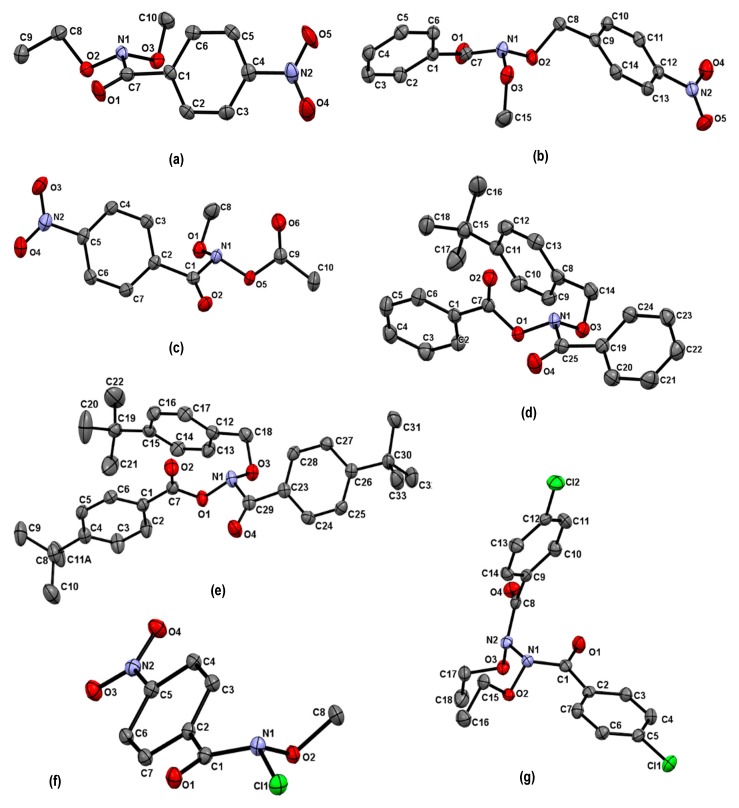
X-ray structures for (**a**) *N*-ethoxy-*N*-methoxy-4-nitrobenzamide **5a** [33], (**b**) *N*-methoxy-*N*-(4-nitrobenzyloxy)benzamide **5b** [33], (**c**) *N*-acetoxy-*N*-methoxy-4-nitrobenzamide **7**[65], (**d**) *N*-benzoyloxy-*N*-(4-*tert*-butylbenzyloxy)benzamide **6b** [29], (**e**) *N*-(4-*tert*-butylbenzoyloxy)-*N*-(4-*tert*-butylbenzyloxy)-4-*tert*-butylbenzamide **6a** [29], (**f**) *N-*chloro-*N*-methoxy-4-nitrobenzamide **9** [65], and (**g**) *N*,*N*′-4-chlorobenzoyl-*N*,*N*′-diethoxyhydrazine **8** [32].

The unusual structure of a number of stable anomeric amides (**5**–**9**) has been confirmed by X-ray crystallography (Figure 6) and relevant structural parameters of these are given in Table 1. The X-ray structures of anomeric amides **5**–**9** provide clear evidence of reduced amide resonance. The data in Table 1 shows that as the combined electron demands of X and Y increase, N–C(O) bond length and pyramidalisation at nitrogen both increase. The reported average N–C(O) bond length in acyclic amides is 1.359 Å (median 1.353 Å), generated from Cambridge Structural Database (CSD) [29,63], which is significantly shorter than the average 1.418 Å from these seven X-ray structures. While the (N)C=O bond (average 1.207 Å) contracts slightly compared to simple acyclic amides (1.23 Å), there is little to no correlation seen between change in (N)C=O bond length and degree of lone pair dislocation; this may be attributed to bias towards carbon in the LUMO (Figure 1) [2,26,35,64]. Deviation from the usual sp^2^ hybridisation at the amide nitrogen (χ_N_ = 0) is significant and in line with the electron demands of substituents. *N*-acyloxy-*N*-alkoxyamides **6a**, **6b**, and **7** (χ_N_ = 65.33°, 65.62° and 59.7°, respectively) are pyramidalised at nitrogen to the extent of, and beyond, what is expected for a pure sp^3^ hybridisation. The 4-nitrobenzamide **7** is less pyramidal than the benzamides **6a** and **6b** presumably on account of greater positive charge at the carbonyl carbon and attendant increase in nitrogen lone pair attraction. Both *N*,*N*-dialkoxyamides **5a** and **5b** (χ_N_ = 58.3° and 55.6°, respectively) are also strongly pyramidalised. For Shtamburg’s *N*-alkoxy-*N*-chloroamide **9**, X-ray data reveals a small χ_N_ of 52.5°, similar to *N*,*N*-dialkoxyamides **5a** and **5b**. Both amide nitrogens in hydrazine **34** are the least pyramidalised with χ_N__1_ and χ_N__2_ of 47° and 49°, respectively.

Despite high degrees of pyramidalisation in this set of anomeric amides, there is minimal twist about the N–C(O) bond (τ = 6.7–15.5°). This indicates that lone pair orbital overlap with the carbonyl C2p_z_ orbital, though clearly less effective on electronic and geometric grounds, remains a stabilizing influence (Table 1). As can be seen in the B3LYP/6-31G(d) deformation surface for *N*,*N*-dimethoxyacetamide **4b** (Figure 5b), the strongly pyramidal structure requires twist angles (τ) beyond 20° before there is significant loss of stabilization.

Pyramidal nitrogens and corresponding anomeric interactions have also been observed in the structures of a number of urea and carbamate analogues of anomeric amides (**10**–**14**) studied by Shtamburg and coworkers and selected structural data are presented in Table 2 [66,67,68,69]. Where substituent electronic effects are largely similar as in **11b** and **13**, it can be deduced that the stronger conjugative effect of the α-nitrogen lone pair in the urea **11b,** relative to the α-oxygen in the carbamate **13,** results in significantly less amide resonance interaction and, hence, significant increase in pyramidality at the amide nitrogen. Comparison of the ONCl structures **9** and **10a** and **b**, again indicates greater pyramidality in the ureas where competing acyl nitrogen resonance reduces the anomeric amide resonance interaction.


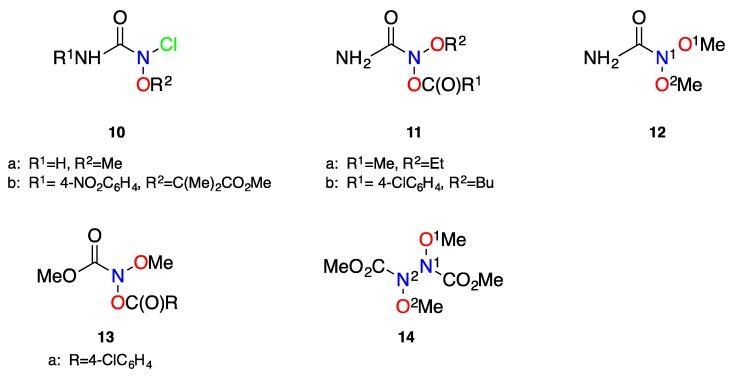


**Figure 7:**
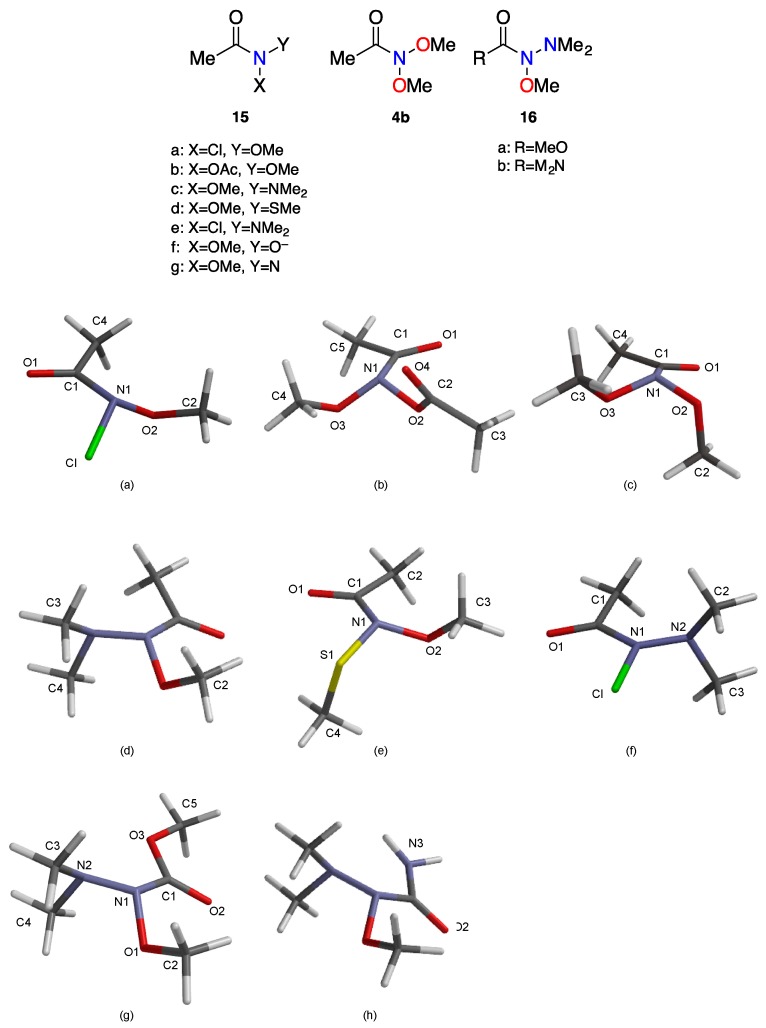
B3LYP/6-31G(d) lowest energy ground state conformers of model anomeric acetamides (**a**) *N*-chloro-*N*-methoxyacetamide **15a,** (**b**) *N*-acetoxy-*N*-methoxyacetamide **15b**, (**c**) *N,N*-dimethoxyacetamide **4b**, (**d**) *N*-methoxy-*N*-dimethylaminoacetamide **15c**, (**e**) *N*-methoxy-*N*-methylthiylacetamide **15d**, (**f**) *N*-chloro-*N*-dimethylaminocetamide **15e**, (**g**) *O*-methyl *N*-methoxy-*N*-dimethylaminoacetamide **16a**, and (**h**) *N*-methoxy-*N*-dimethylaminourea **16b**.

These and other anomeric amides have been modelled at B3LYP/6-31G(d) level in the simplified acetamide system and ground state models of **15a**–**d** systems display high degrees of pyramidality, little N–C(O) twist, long N–C(O) bonds, and slightly shortened (N)C=O bonds, in line with the electron demands of substituents and, where applicable, are reasonable approximations of their respective X-ray structure counterparts (Figure 7, Table 3). ONS and NNCl analogues, **15d** and **15e**, have only been generated as intermediates in reactions but their theoretical structures are in line with those of anomeric systems **15a**–**d**; N–C(O) bond lengths and pyramidality at nitrogen (χ_N_) are broadly in line with the gross electronegativity of substituents at nitrogen. Sulphur, with its low electronegativity, results in a less pyramidal nitrogen, while the NNCl system **15e** is completely planar (and untwisted) for steric reasons. Nonetheless, its N–C(O) bond is comparatively long. The carbamate and the urea **16a** (χ_N_ = 46.3°) and **16b** (χ_N_ = 49.6°) are both more pyramidal than the corresponding acetamide **15c** (χ_N_ = 41.8°), presumably as a result of competing resonance from the α-oxygen and α-nitrogen lone pairs.

### 2.2. Resonance Energies and Amidicities

The resonance energy and the amidicity of anomeric amides **4b** and **15a**–**e** have been calculated at the B3LYP/6-31G(d) level by both the TA and COSNAR methods and by the COSNAR method using dispersion corrected M06/6-311++G(d,p). Δ*E***_COSNAR_** (Equation (1)), reaction energies (Δ*E*_TA_) (Equation (2)), inductive destabilization corrections (Δ*E***_ind_**) (Equation (3)), resultant RE**_TA_** (Equation (4)) together with COSNAR, and TA amidicities are presented in Table 4.

The TA and COSNAR methodologies give almost identical resonance energies for all the anomeric amides (**4b** and **15a**–**e**) at the B3LYP/6-31G(d) level. Δ*E***_COSNAR_**determined at M06 with the expanded basis set yields very similar results to B3LYP/6-31G(d) for *N*,*N*-dimethylacetamide **1** and the anomeric amides with the exception of **15e**, where resonance is computed to be worth 49 kJ mol^−1^, just over 60% that of *N,N-*dimethylacetamide and substantially higher than that predicted from B3LYP/6-311++G(d,p), which was identical to the B3LYP/6-31G(d) value (29 kJ mol^−1^) [34]. The RE parity for **15a** and **15e** at B3LYP is no longer observed with M06, in line with the lower overall electronegativity of nitrogen and chlorine. These results impute the necessity for inclusion of dispersion corrections in treatments of molecules where anomeric interactions are likely to have a pronounced influence.

It is clear that resonance in anomeric amides is impaired, broadly in line with the gross electronegativity or negative inductive effect of the atoms/groups bonded to nitrogen. Interestingly, the ONN and ONS acetamides, **15c** and **15d**, have very similar resonance energies despite the much lower electronegativity of sulphur. From Bent’s rule, the opposite effect would be expected, since s-character in the lone pair orbital should decrease with decreasing electronegativity of X. This is likely to be a manifestation of the role of orbital size since the influence of second period elements is significant; nitrogen increases p-character in the bond to sulphur to effect better overlap with the larger, 3p orbital of sulphur. Consequently, the nitrogen lone pair gains more “s” character relative to the amide nitrogen in the NNO acetamide **15c**, resulting in less amide resonance [36,37,70]. Comparing the M06 values, ONCl and ONO systems have about half the resonance of *N*,*N*-dimethylacetamide while NNO, NNCl, and ONS systems are computed to preserve some 60 to 70% of the resonance of *N*,*N*-dimethylacetamide.

### 2.3. The Anomeric Effect

**Figure 8:**
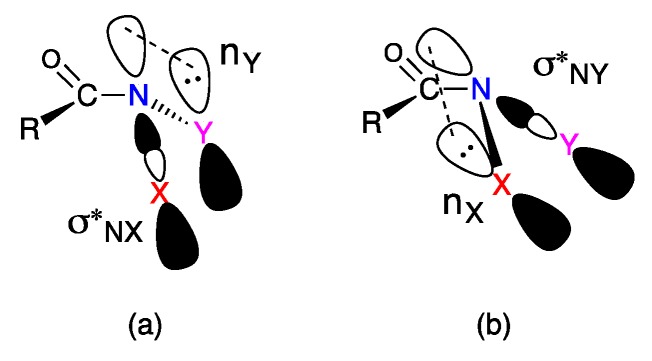
Two possible anomeric interactions in anomeric amides (**a**) n_Y_–σ*_NX_ and (**b**) n_X_–σ*_NY_.

**Figure 9:**
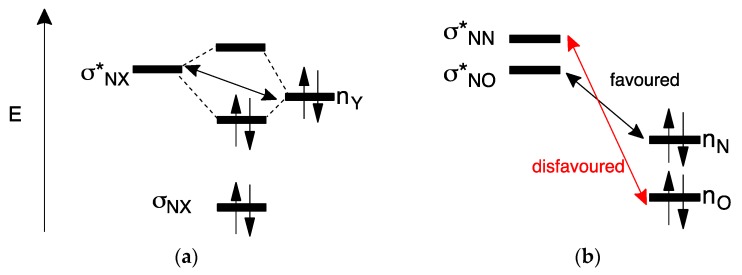
(**a**) Stabilisation of a lone pair through a n_Y_–σ*_NX_ anomeric interaction and (**b**) the energetics of the anomeric effect in **15c**.

In addition to their reduced amide resonance, anomeric amides are exemplars of XNY systems featuring an anomeric effect [26,35]. There are two possible anomeric interactions designated as n_X_–σ*_NY_, and n_Y_–σ*_NX_ and where X and Y are different electronegative atoms, one of these interactions will be favoured over the other (Figure 8). By analogy with anomeric carbon centres [26,35,71,72,73], the relative electronegativities of heteroatoms X and Y at nitrogen and the relative sizes of interacting orbitals contribute to the strength of an anomeric interaction. Heteroatoms Y and X directly influence the relative energies of n_Y_ and σ*_NX_, which, in turn, affect the net stability gain for the lone pair electrons (Figure 9a).

**Figure 10:**
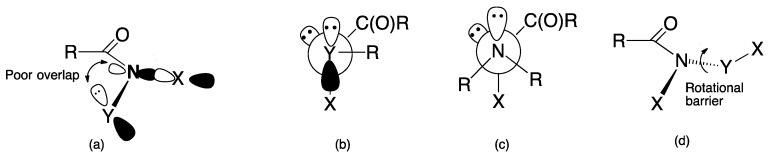
(**a**) sp^2^ hybridised nitrogen hinders overlap with heteroatom Y in an n_Y_–σ*_NX_ system; (**b**) optimum conformation in an n_Y_–σ*_NX_ anomeric interaction; (**c**) optimum conformation in an n_N_–σ*_NX_ anomeric interaction; and (**d**) restricted rotation about the N–Y bond in an n_Y_–σ*_NX_ anomeric interaction.

As the electronegativity of X and Y increases by going across the p-block row on the periodic table, σ*_NX_ (and σ_NX_) decreases in energy as does n_Y_. Additionally, as X decreases in electronegativity by going down a p-block group, σ*_NX_ decreases in energy due to reduced orbital overlap [26,35,36,71,72]. An optimal anomeric effect can be achieved when Y is an early p-block element and X is a p-block element to the right of Y on the periodic table; more specifically an anomeric stabilisation will be greater when the energy gap between n_Y_ and σ*_NX_ is lower (Figure 9b) [74,75]. In the unusual case of anomeric amides, where the nitrogen may range between planar sp^2^ and pyramidal sp^3^ hybridisation, the geometry of the central nitrogen atom in XNY systems also plays a role, as pyramidal nitrogen is more conducive to edge-on n_Y_ and σ*_NX_ overlap than is planar, sp^2^ hybridised nitrogen (Figure 10a) [26,35].

Similar to XCY configurations, in an XNY system, a stabilising n_Y_–σ*_NX_ anomeric effect, for example, can cause the amide to adopt a gauche conformation in which the lone pair of Y is coplanar with the vicinal to C–X bond (Figure 10b) [71,74]. In anomeric amides, when n_Y_ is divalent oxygen or sulphur, an R-Y-N-X dihedral angle close to |90°| aligns the p-type lone pair on those atoms with the vicinal σ*_NX_ (Figure 10b). Where the donor is n_N_, an optimum anomeric effect would need the lone pair on nitrogen to be antiperiplanar to X, LP(N)-N-N-X = 180° (Figure 10c). Consequences of the n_Y_–σ*_NX_ interaction include an increased barrier to rotation about the N–Y bond (Figure 10d), a contraction of the N–Y bond, and an extension of the N–X bond as electrons from Y populate the σ*_NX_ orbital.

In each of the X-ray structures in Figure 6 there is clear evidence of these anomeric interactions (Table 1, bold-face torsion angles). On the basis of energetics, the expected anomeric interactions are n_O_–σ*_NOAc_ in **6a**, **6b**, and **7**, n_N_–σ*_NO_ in **8**, and n_O_–σ*_NCl_ in **9**. In **5a** and **5b** one n_O_–σ*_NO_ would be expected to prevail. Dihedral angles about the N–O bonds in **5**–**7** and **9** show a preferred p-type lone pair alignment with the adjacent σ*_NO_, σ*_NOAc_, or σ*_NC__l_ bond. In hydrazine **8**, the lone pair on N1 is almost perfectly aligned with the N2–O3 bond while the N2 lone pair makes an angle of only 47° to the N1–O2 bond (1.403 Å), which is shorter than the anomerically destabilised bond N2–O3 (1.411 Å). The structure is asymmetrical with a donor N1 and recipient N2. Similar anomeric interactions are observable in ureas **10**–**12** and carbamates **13** and **14** (Table 2).

All computed structures exhibit an anomeric interaction (Table 3, bold-face torsion angles) and, besides the ONO system where one n_O_–σ*_NO_ prevails, an anomeric n_O_–σ*_NX_ prevails in ONCl, ONOAc, and ONS in line with expectations based on electronegativity. The size of the sulphur p-orbital and lower energy of the σ*_NS_ renders an n_S_–σ*_NO_ anomeric interaction less likely than a n_O_–σ*_NS_ stabilisation. Where nitrogen is present, the n_N_–σ*_NO_ and n_N_–σ*_NCl_ interactions are clearly in evidence.

The anomeric effects not only dictate stereochemistry at nitrogen but, as will be seen, combined with reduced amide resonance, they have a profound impact upon spectroscopic properties and the reactivity of anomeric amides.

### 2.4. Spectroscopic Properties of Anomeric Amides

Spectroscopic properties of anomeric amides are strongly influenced by reduced resonance due to electronegativity of substituents at nitrogen. Infrared and ^13^C NMR data for a diverse range of ONCl, ONOAcyl [26,31,35], and ONO systems [35,55] have been reported as well as for a number of ONN *N*,*N′*-dialkoxy-*N*,*N′-*diacylhydrazines [76,77,78]. Representative infrared carbonyl stretch frequencies in solution for stable anomeric amides (Table 5) are significantly higher (1700 to 1750 cm^−1^) [32,47,55,79,80,81] than those of their precursor hydroxamic esters (1650 to 1700 cm^−1^) and primary (1690 cm^−1^), secondary (1665 to 1700 cm^−1^), and tertiary (1630 to 1670 cm^−1^) alkylamides [26,35,82]. While there is a slight tightening of the (N)C=O bond, the increase in ν_C=O_ has been attributed primarily to electronic destabilisation of single-bond resonance form of the carbonyl as electron density is pulled towards the electronegative heteroatoms on nitrogen, resulting in a more ketonic carbonyl bond [2,64]. Likewise, anomeric amides exhibit more ketonic carbonyl ^13^C chemical shifts (CDCl_3_), with downfield shifts of approximately 8.0 ppm from their hydroxamic ester precursors. Deshielding of the carbonyl carbon is an expected consequence of the electron-withdrawing substituents. However, like acid chlorides and anhydrides, the carbonyls resonate upfield of ketones, relative to which the electron density at the carbonyl is increased on account of greater double bond character.

Common primary, secondary, and tertiary amides have significant *cis–trans* isomerisation barriers for rotation about the N–C(O) bond, due to the stabilising effect of amide resonance in their ground state [2]. Restricted rotation through lone pair overlap with the adjacent carbonyl C2p_z_ orbital, as illustrated in Figure 1, often results in observation of different chemical shifts of *cis* and *trans* conformers in their ^1^H NMR spectra, from which barriers to isomerisation can be deduced [83,84]. The computed surface for *N*,*N*-dimethylacetamide in Figure 3 indicates that the barrier (difference between the completely planar and the fully rotated-pyramidal forms) is of the order of 71–75 kJ mol^−1^. Hydroxamic esters have slightly less resonance and amidicity, and the rotation barrier for *N*-methoxy-*N*-methylacetamide from Figure 5a is approximately 67 kJ mol^−1^. Many hydroxamic esters have broadened ^1^H NMR signals at ambient temperatures. Anomeric amides with lower resonance should have lower *cis*–*trans* isomerisation barriers as exemplified in the model *N*,*N*-dimethoxyacetamide in Figure 5b. Accordingly, the isomerisation barrier in bisoxyl-substituted amides **2b** and **2c** are too low to be measured by usual dynamic NMR methods. All proton signals of *N*,*N*-dimethoxy-4-toluamide remained sharp down to 180 K (in *d*_4_-methanol) and the ^1^H NMR signals for *N*-acetoxy-*N*-benzyloxybenzamide remained isochronous down to 190 K (in *d*_8_-toluene) [26]. The barrier for *N*-benzyloxy-*N*-chlorobenzamide **18** could likewise not be determined by dynamic NMR [35].

**Figure 11:**
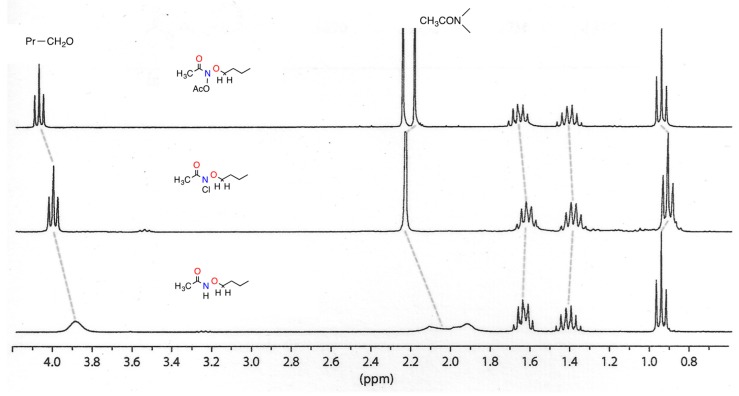
300 MHz ^1^H NMR spectra (300K, CDCl_3_) of *N*-butoxyacetamide, and anomeric amides *N*-butoxy-*N*-chloroacetamide and *N*-acetoxy-*N*-butoxyacetamide.

Figure 11 illustrates the dramatic difference between ^1^H NMR spectra of *N*-butoxyacetamide and its *N*-chloro- or *N*-acetoxyl derivatives as a consequence of reduced resonance in the anomeric structures, which clearly have much lower isomerization barriers. At room temperature, the anomeric amides are in the fast exchange region as opposed to the slow exchange in the hydroxamic ester. Earlier theoretical studies by Glover and Rauk support this assertion [27,28]. For example, a comparison of formamide **17a**, *N-*methoxyformamide **17b**, and *N*-chloro-*N*-methoxyformamide **17d**, calculated at the B3LYP/6-31G(d) level, showed little reduction in the *cis*–*trans* isomerisation barrier of 73.2 kJ mol^−1^ in formamide, to 67–75 kJ mol^−1^ in *N*-methoxyformamide. However, the introduction of a second electronegative heteroatom, Cl, reduced the barrier to only 32.2 kJ mol^−1^, indicating that monosubstitution alone is insufficient to impact upon the isomerisation barrier [27]. A theoretical isomerisation barrier for *N*-methoxy-*N*-(dimethylamino)formamide, which, by analogy with typical NNO systems, should retain ~70% the resonance in *N*,*N*-dimethylacetamide, was computed to be higher at 52.7 kJ mol^−1^ [28]. Such an amide isomerisation barrier in the hydrazine, *N*,*N′*-diacetyl-*N*,*N′*-di(4-chlorobenzyloxy)hydrazine **20d**, was measurable at Δ*G*^‡^_278_ *=* 54.0 kJ mol^−1^, in line with this theoretical barrier [27].

The intrinsic barrier to inversion at nitrogen in bisheteroatom-substituted amides is expected to be much lower than those of analogous amines. The transition state for inversion in anomeric amides is expected to be planar, where the nitrogen lone pair can interact with the carbonyl 2p_z_ orbital generating stabilisation [35]. For *N*,*N*-dimethoxyacetamide (Figure 5b) this barrier is represented by the energy of structure (b), while the difference in energy between fully twisted structures (d) and (c) represents the much larger barrier to inversion in the corresponding anomeric amine. Several theoretical estimates put these barriers in anomeric amides at about 10 kJ mol^−1^ [28].


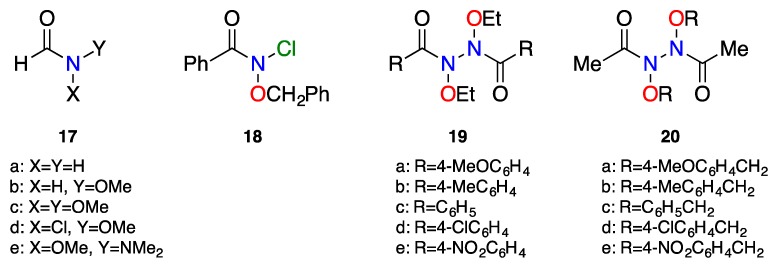


In anomeric systems, strong n_Y_–σ*_NX_ interactions should increase the bond order of the N–Y bond, which should impose barriers to rotation about those bonds. For *N*-chloro-*N*-methoxyformamide **17d**, the N–O and N–C(O) rotation barriers have been estimated at the B3LYP/6-31G(d) level at 44.7 and 29.2 kJ mol^−1^, respectively [27], while the theoretical barrier to rotation about the N–N bond in *N*-methoxy-*N*-dimethylaminoformamide **17e** was computed at ~60 kJ mol^−1^ [28]. Experimental measurements for both anomerically induced barriers have been made. An N–O anomeric rotational barrier of ∆*G*^‡^ = 43 kJ mol^−1^ has been determined for *N*-chloro-*N*-benzloxybenzamide **18**, where the benzylic methylenes become diastereotopic at 217 K in *d*_8_-toluene, but as no amide isomerisation could be detected, that barrier must be significantly lower [26]. An anomerically induced rotational barrier for N–N′ bond in a number of *N*,*N*-diacyl-*N*,*N*-dialkoxyhydrazines **19a**–**e** and **20a**–**e** has been measured in dynamic ^1^H NMR studies [32]. Methylene signals of *N*,*N*′-diethoxy **19a**–**e** and *N*,*N*′-dibenzyloxy groups **20a**–**e**, which were diastereotopic at room temperature, as a consequence of restricted rotation about the N–N′ bond, coalesced at higher temperatures (T_c_ = 316–346 K) from which rotational free energy barriers of the order of 60−70 kJ mol^−1^ could be determined [32], which compare favourably with the theoretically calculated *N*–*N*′ rotational barrier for **17e** of 60 kJ mol^−1^ [28].

## 3. Reactivity of Anomeric Amides

The reduced resonance and attendant pyramidalisation at the amide nitrogen together with anomeric properties of these unusual molecules results in a plethora of amide reactivity, some known, and now better understood, and others that are unique to anomeric amides. The destabilisation of the amide bond, coupled with the substitution pattern, facilitates reactivity at the amide nitrogen in which the amides are usually transformed from one anomeric amide form to another. Moreover, it can induce a novel process, known as the HERON reaction (Named at the Third Heron Island Conference on Reactive Intermediates and Unusual Molecules, Heron Island 1994.), in which the amide bond is broken to form acyl derivatives and heteroatom-stabilised nitrenes. This reaction is facilitated by weakened amide resonance, but is driven by n_Y_–σ*_NX_ anomeric destabilisation of the N–X bond.

### 3.1. Reactivity at the Amide Nitrogen

Due to their characteristic, diminished amide resonance and anomeric destabilisation, this class of amide has been shown to undergo S_N_2 reaction at nitrogen, and elimination of *N*-substituents leading to S_N_1-type processes. Several congeners undergo thermolytic homolysis to give alkoxyamidyl radicals.

#### 3.1.1. S_N_2 Reactions

**Scheme 1:**
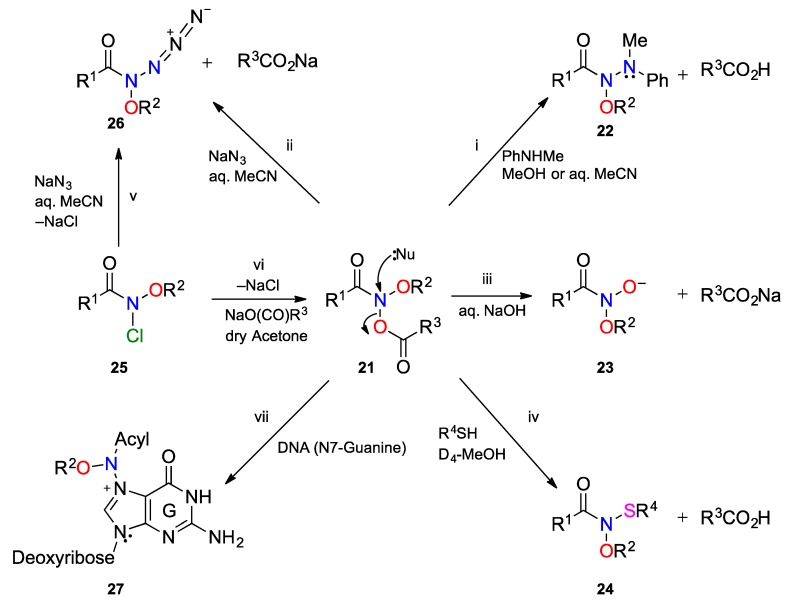
S_N_2 reactions of *N*-acyloxy-*N*-alkoxyamides **21** with: (i) arylamines; (ii) azide; (iii) hydroxide; (iv) thiols; (vii) DNA and reaction of *N*-alkoxy-*N*-chloroamides **25** with (v) azide and (vi) sodium carboxylates.

In XNY systems, a moderate n_Y_–σ*_NX_ negative hyperconjugation leads, through neighbouring group participation, to weakening of the N–X bond, which can encourage S_N_2 reactions at nitrogen [26,31,35]. The increased electrophilicity of nitrogen in *N*-acyloxy-*N*-alkoxyamides **21** leaves them vulnerable to attack by arylamines (Scheme 1 i) [42,44,47,51], azide (Scheme 1 ii) [54], hydroxide (Scheme 1 iii) [45], and thiols (Scheme 1 iv) [52], the outcomes from which are anomerically substituted intermediates **22**–**24** and **26** that ultimately may undergo HERON reactions. Furthermore, *N*-acyloxy-*N*-alkoxyamides **21** themselves may be synthesised in S_N_2 reactions between *N*-alkoxy-*N*-chloroamides **25** and sodium carboxylates (Scheme 1 vi) [43,45,46,48,51,80,85]. *N*-alkoxy-*N*-chloroamides **25** also react bimolecularly with azide generating reactive *N*-alkoxy-*N*-azidoamides **26** (Scheme 1 v) [54].

**Figure 12:**
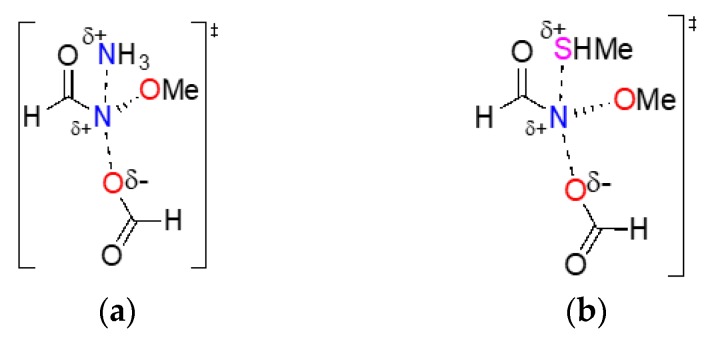
HF/6-31G* predicted charge separation in transition states for reactions of *N*-formyloxy-*N*-methoxyformamide with (**a**) ammonia and (**b**) methanethiol.

Reactions of **21** systems with amines and thiols have been modelled at the AM1, HF/6-31G(d), and pBP/DN* levels, which reveal significant charge separation in the transition states and alkoxynitrenium ion character (Figure 12) [86]. These reactions should be favoured by electron-donor substituents on the nucleophile and electron-acceptor substituents on the acyloxyl group.

The S_N_2 reaction of *N*-methylaniline with a wide range of *N*-acyloxy-*N*-alkoxyamides **21** has been studied (Scheme 1 i), and relative rate constants, Arrhenius activation energies, and entropies of activation are in accord with a transition state with significant charge separation [31,44,51,87]. *E*_A_’s are of the order of 40 to 60 kJ mol^−1^. Entropies of activation (−90–160 J K^−1^ mol^−1^) are more negative than found in S_N_2 reactions of alkyl halides, owing to a greater degree of solvation in the charge separated transition state [88]. In addition, the rates for reactions i, iii, and iv with a series of NAA’s bearing *N*-*p*-substituted benzoyloxyl leaving groups correlated with Hammett σ constants with positive slope (i, ρ = 1.7, iii, ρ = 0.6, and iv, ρ = 1.1) [44,45,52]. In addition, a series of anilines reacted bimolecularly and rate constants correlate with Hammett σ^+^ constants (ρ = −0.9) [44]. In most respects, the S_N_2 reactions are electronically and geometrically reminiscent of those at carbon centres and are accelerated by electron-donor groups on the nucleophile and electron-withdrawing groups on the leaving group. The amide carbonyl facilitates S_N_2 reactivity in line with enhanced reactivity in phenacyl bromides [89]. In particular, bimolecular reaction rates are radically impeded with branching α to the carbonyl [50], which is analogous to the resistance to S_N_2 reactions of α-halo ketones bearing substituents at the α’ position [90,91].

Anomeric substitution at nitrogen in *N*-acyloxy-*N*-alkoxyamides **21** renders this class of amides as direct-acting mutagens. Mutagenicity towards *S. typhimurium* in the Ames reverse mutation assay does not require premetabolic activation [92,93]. Our DNA damage studies on plasmid DNA at physiological pH, as well as extensive structure–activity relationships [31,38,39,40,41,43,45,46,48,49,50,79,80], point to binding of NAA’s **21**, intact, into the major groove of DNA, where an S_N_2 reaction occurs at the most nucleophilic centre, the electron-rich N7 in guanine (Scheme 1 vii) [94,95,96,97]. All three side chains (R^1^, R^2^, and R^3^) of **21** have an impact upon both DNA damage profiles as well as mutagenicity levels. An S_N_1 mechanism, yielding electrophilic *N*-acyl-*N*-alkoxynitrenium ions, was ruled out since only R^1^ and R^2^ would influence binding and reactivity. Moreover, mutagenic activity is radically reduced when there is branching α to the carbonyl in parallel with the impaired S_N_2 reactivity [40,50,51]. The mutagenic activity of *N*-acyloxy-*N*-alkoxyamides **21** has been used recently to show how hydrophobicity and intercalating side chains impact upon DNA binding [38,39].

#### 3.1.2. Elimination Reactions

**Scheme 2:**
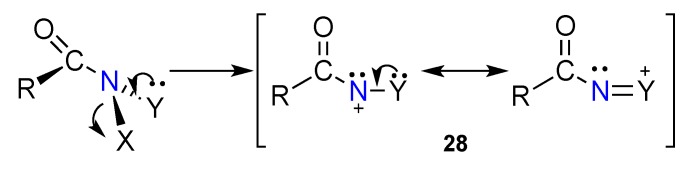
Nitrenium ion formation by elimination of X due to a strong n_Y_–σ*_NX_ interaction.

**Scheme 3:**
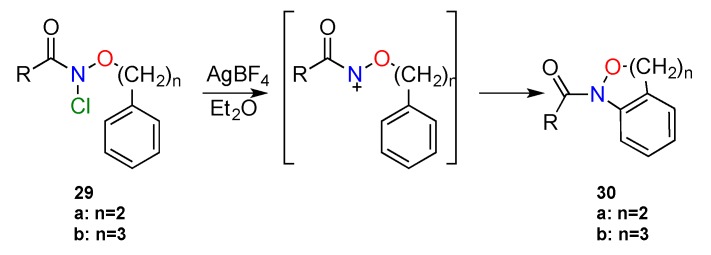
Cyclisation by silver ion catalysed elimination reactions.

In an anomeric amide where n_Y_–σ*_NX_ is a strong interaction, where X has a high electron affinity and Y is a strong electron donor, polarisation can lead to elimination of X^−^, leaving a Y-stabilised nitrenium ion **28** (Scheme 2) [98]. The stronger the anomeric effect, the more readily the elimination is expected to occur. In the case of *N*-alkoxy-*N*-chloroamides **25**, elimination can be facilitated by Lewis acid complexation with X and by the use of polar solvents [35]. For example, treatment of *N*-chloro-*N*-(2-phenylethyloxy)- **29a** and *N*-chloro-*N*-(3-phenylpropyloxy)amides **29b** with silver tetrafluoroborate in ether, initiates a ring closing reaction to form *N*-acyl-1*H*-3,4-dihydro-2,1-benzoxazines **30a** and *N*-acyl-1,3,4,5-tetrahydrobenzoxazepines **30b**, respectively, via chlorine elimination to form nitrenium ions (Scheme 3). *N*-acyl-*N*-alkoxynitrenium ions are strongly stabilised by delocalisation of the positive charge onto oxygen [98,99]. This methodology has been used widely since its discovery in 1984 by Glover [100,101] and Kikugawa [102,103,104,105,106,107,108]. In addition, treatment of *N*-alkoxy-*N*-chloroamides **25** with silver carboxylates in diethyl ether, allows the nitrenium ion to be scavenged by carboxylate in a versatile reaction which has been used to synthesise a range of *N*-acyloxy-*N*-alkoxyamides **21** [48,80].

**Scheme 4:**
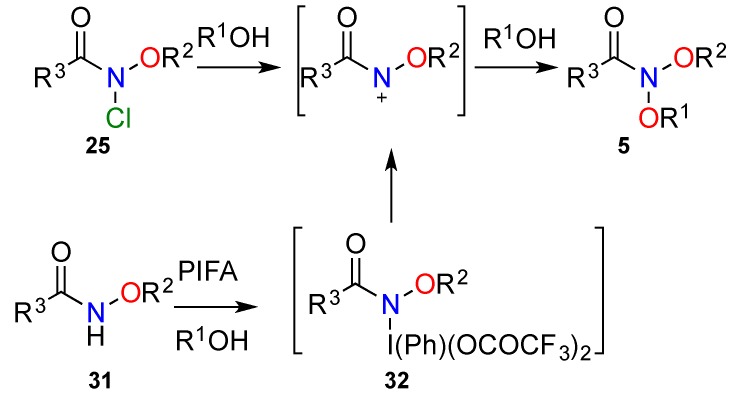
*N*-Acyl-*N*-alkoxynitrenium ion mediated syntheses of *N*,*N*-dialkoxyamides **5**.

Elimination of chloride in the alcoholysis of **25** to give nitrenium ion provided a synthetic pathway to *N*,*N*-dialkoxyamides **5** (Scheme 4) [55,56]. We recently reported a more versatile synthesis effected by PIFA oxidation of hydroxamic esters **31** in appropriate alcohol, which proceeds through a reactive phenylbistrifluoroacetate derivative **32** [30,55]. Similar hypervalent iodine oxidations have been used in nitrenium ion cyclisations onto aromatic rings [105].

**Scheme 5:**
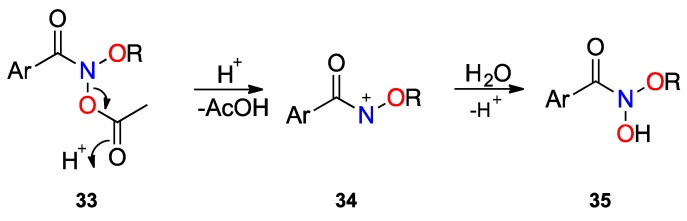
Acid catalysed hydrolysis of *N*-acetoxy-*N*-butoxybenzamides.

*N*-Alkoxy-*N*-benzoylnitrenium ions **34** are generated through A_A1_1 acid-catalysed solvolysis of *N*-acetoxy-*N*-alkoxybenzamides **33** (Scheme 5) [46,48,49]. Acetoxyl, upon protonation with a catalytic amount of mineral acid, is eliminated from *N*-acetoxy-*N*-alkoxybenzamides **33** and the nitrenium ions are trapped by water to form *N-*alkoxyhydroxamic acids **35**. The anomeric **35** undergoes secondary reactions to form a range of products.

The elimination reactions of *N*-alkoxy-*N*-chloroamides **25** and the acid-catalysed solvolysis reactions of **33**, both of which proceed through intermediacy of *N*-acyl-*N*-alkoxynitrenium ions, can better be re-evaluated in terms of anomeric destabilisation in combination with their reduced amidicities.

### 3.2. The HERON Reaction

#### 3.2.1. HERON Reactions of *N*-Amino-*N*-Alkoxymides

**Scheme 6:**
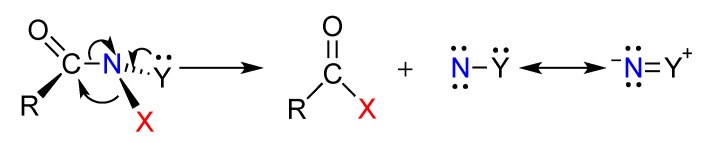
The heteroatom rearrangement on nitrogen (HERON) reaction of an anomeric amide.

A novel reaction of suitably constituted anomeric amides is the HERON (Heteroatom Rearrangement On Nitrogen) reaction [56,109,110,111,112,113]. In such amides, when the X heteroatom of an anomerically destabilised N–X bond is a poor leaving group, the amide can undergo a concerted rearrangement involving the migration of X to the carbonyl carbon and the ejection of a Y-stabilised nitrene (Scheme 6).

**Scheme 7:**
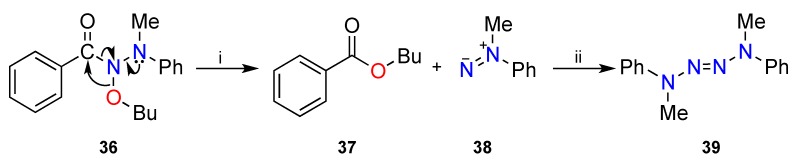
The first HERON reaction of *N*-butoxy-*N*-(*N*′-methylanilino)benzamide **36**.

**Scheme 8:**
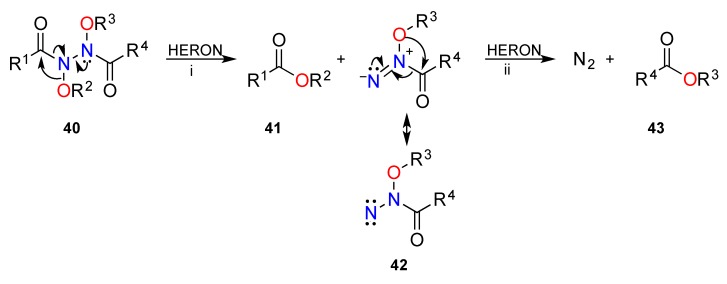
Tandem HERON reactions of *N*,*N*′-diacyl-*N*,*N*′-dialkoxyhydrazines **40**.

The HERON reaction was discovered by Glover and Campbell during research into S_N_2 reactivity of *N*-acyloxy-*N*-alkoxyamides **21**, specifically the reaction between *N*-acetoxy-*N*-butoxybenzamide and *N*-methylaniline according to Scheme 1 i [47,111]. In a polar solvent such as methanol, *N*-methylaniline attacks the amide nitrogen, replacing the acetoxyl side chain to form an unstable intermediate, *N*-butoxy-*N*-(*N*′-methylanilino)benzamide **36**, which undergoes the HERON reaction to form butyl benzoate **37**, and an aminonitrene, 1-methyl-1-phenyldiazene **38** (Scheme 7 i). Aminonitrenes are highly reactive intermediates with a singlet ground state, which persist long enough under reaction conditions to dimerise to tetrazenes [114,115,116,117], in this case, **39** (Scheme 7 ii) [44,47]. *N*,*N*′-Diacyl-*N*,*N*′-dialkoxyhydrazines **40**, the only forms of *N*-alkoxy-*N*-aminoamides **2d** to have been isolated, undergo tandem HERON reactions to form two equivalents of ester **41** and a molecule of nitrogen; the *N*-acyl-*N*-alkoxyaminonitrenes **42**, formed in this HERON reaction, rapidly undergo a second rearrangement to form a molecule of nitrogen and ester before dimerisation of the *N*-acyl-*N*-alkoxyaminonitrene can occur (Scheme 8) [56,76]. Step ii can also be regarded as a HERON process, driven by a high energy electron pair on 1,1-diazene, a charge separated form of aminonitrene. Barton and coworkers studied the decomposition at about the same time, and both groups established the operation of three-centre mechanisms using asymmetric hydrazines [77]. In addition, Barton found its concerted nature facilitated the formation of a range of highly hindered esters and, recently, Zhang has utilised the reaction to generate hindered esters from *N*,*N*′-dialkoxy-*N*,*N*′-diacylhydrazines, synthesised through *N*-bromosuccinimide (NBS) oxidation of hydroxamic esters [118].

#### 3.2.2. Theoretical and Experimental Validation of the HERON Reaction

**Figure 13:**
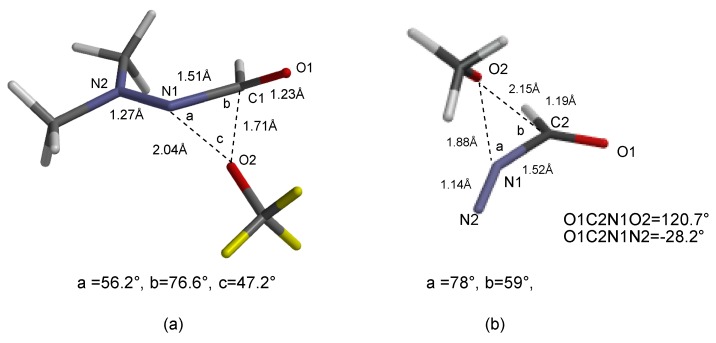
Twisted HERON transition states of (**a**) *N*-methoxy-*N*-dimethylaminoformamide **17e** and (**b**) 1-formyl-1-methoxydiazene **44** (R^1^ = H, R^2^ = Me) at B3LYP/6-31G(d) level [53,76].

The HERON reaction of **22** and **40** has been modelled and validated computationally. Initially, AM1 modelling predicted that the three-centre reaction in the first HERON (Scheme 7 i) had an energy barrier of 184 kJ mol^−1^, while that of the second step was very low (25 kJ mol^−1^) [56,77]. An extensive AM1 study of HERON reactions of *N*-amino-*N*-alkoxyacetamides predicted a similar barrier of 159 kJ mol^−1^ in the gas phase, but a lower barrier of 126 kJ mol^−1^ in solution [111]. The same study also predicted lower barriers with electron-donor groups on the amino nitrogen. In a more rigorous study at the B3LYP/6-31G(d) level [76], Glover et al. modelled the HERON reaction of *N*-methoxy-*N*-dimethylaminoformamide **17e**, a model representative of *N*-(*N*′-methylanilino)-*N*-butoxybenzamide **36**, and *N*,*N*′-diacyl-*N*,*N*′-dialkoxyhydrazines **40**, for which HERON reactions had been experimentally observed. It was confirmed that the n_N_–σ*_NO_ anomeric destabilisation resulted in migration of the methoxyl group with an activation barrier of 90 kJ mol^−1^ and the reaction was exothermic by 23 kJ mol^−1^. Similar diasteromeric transition states were located, but the transition state accessible from the lowest energy (*syn*) conformer of **17e** was found to be that depicted in Figure 13a. Importantly, modelling showed that amide resonance in the transition state was largely lost, as migration occurs in a plane perpendicular to the carbonyl, twisting the nitrogen lone pair away from alignment with the π*_C=O_ orbital. Anomeric destabilisation, however, remained along the reaction coordinate, driving the reaction forward. The N–C(O) bond is largely intact at the transition state but breaks as the O2–C1 bond forms in an internal, S_N_2-like reaction at the amide carbon. Significantly, a tetrahedral intermediate is avoided by this process. Subsequent high level calculations on the decomposition of anomeric hydrazines by Tomson and Hall, yielded similar energetics for the HERON process [119].

**Figure 14:**
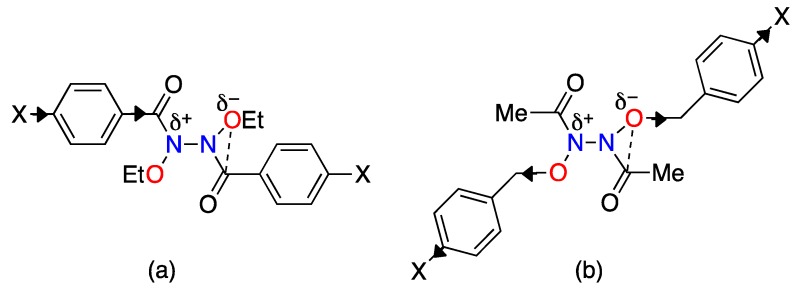
Influence on the HERON transition state of (**a**) electron-rich benzoyl groups in **19** and (**b**) electron-deficient benzyloxy groups in **20**.

Analysis of charge separation in the B3LYP/6-31G(d) transition state revealed a partial positive charge of +0.5 on amino group, partial negative charge of −0.3 on the migrating methoxyl group and little change in charge at the carbonyl. This indicated that HERON in these NNO systems could be assisted by polar solvents, electron-donating groups on the stationary amino substituent, and electron-withdrawing groups on the migrating oxygen substituent. The activation barriers and charge separation in the transition state were validated experimentally by Arrhenius studies and Hammett correlations from thermal decomposition of a range of substituted hydrazines **19a**–**e** and **20a**–**e** in mesitylene (Table 6) [76]. In **19**, the donor ability of n_N_ is increased by electron-rich aroyl groups, leading to enhanced reaction rates and a negative Hammett σ^+^ correlation (ρ = −0.35, R^2^ = 0.978) (Figure 14a). However, acceptor benzyloxy substituents in **20** facilitate the migration, leading to a positive Hammett σ-correlation (ρ = 1.02, R^2^ = 0.911) (Figure 14b). Rate constants were lower in **20** on account of a negative impact at the donor nitrogen. Donor groups, on the other hand, have little impact on the carbonyl in **19**.

#### 3.2.3. HERON Reaction of 1-Acyl-1-Alkoxydiazenes

**Scheme 9:**
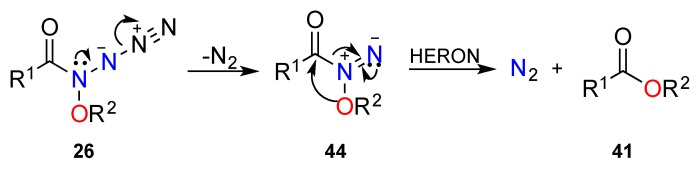
Decomposition and HERON reaction of *N*-alkoxy-*N*-azidoamides **26**.

The second step in the thermal decomposition of *N*,*N*′-dialkoxy-*N*,*N*′-diacylhydrazines **40**, **19**, and **20** has also been modelled at both B3LYP/6-31G(d) and CCSD(T)//B3P86 level using *N*-formyl-*N*-methoxydiazene, and was found to have an extremely small *E*_A_ of between 5 and 12 kJ mol^−1^ and to be highly exothermic (Δ*E* = −400 kJ mol^−1^) [53,119]. We encountered this process from the reaction of *N*-acyloxy-*N*-alkoxyamides **21** with azide (Scheme 1 ii), which generates ester and two molecules of nitrogen [54]. The reaction of *N*-acyloxy-*N*-alkoxyamides with azide was originally conceived in an attempt to trap and determine the lifetimes of *N*-acyl-*N*-alkoxynitrenium ions, by analogy with the determination of lifetimes of arylnitrenium ions in water [120,121]. However, *N*-Alkoxy-*N*-azidoamides **26** are highly unstable intermediates, losing nitrogen to generate 1-acyl-1-alkoxydiazenes **44**, which react further to nitrogen and ester Scheme 9. The transition state for this reaction of *N*-formyl-*N*-methoxydiazene, modelled at the B3LYP/6-31G(d) level, is depicted in Figure 13b. Once again, the methoxyl group migrates in a plane orthogonal to the N1-C2-O1 plane in an earlier transition state with little N–C(O) bond cleavage, which occurs in concert with O2–C2 bond formation, again avoiding a tetrahedral intermediate. The reaction of azide with *N*-chloro-*N*-alkoxyamides **25** proved to be an excellent means of generating highly hindered esters [54]—not surprisingly, in light of the very low *E*_A_ and extreme exothermicity born out of the entropically favourable generation of two highly stable molecules (methyl formate and nitrogen) [53,119]. Overall, the decomposition of *N*-azido-*N*-methoxyformamide to two molecules of nitrogen and methylformate was computed to be exothermic by some 575 kJ mol^−1^ [53]. Yields of esters prepared by this method are given in Table 7.

Both Barton and recently Zhang have shown that the thermal decomposition of *N*,*N*′-dialkoxy-*N*,*N*′-diacylhydrazines is a source of hindered esters. The avoidance of a tetrahedral intermediate in both HERON steps of these reactions, and which is a limiting structure in Fisher esterification, is critical. This, and the clear role of anomeric substitution at nitrogen in both reducing amidicity, as well as promoting the rearrangement, are paramount.

#### 3.2.4. HERON Reactions of Anionic Systems

**Scheme 10:**
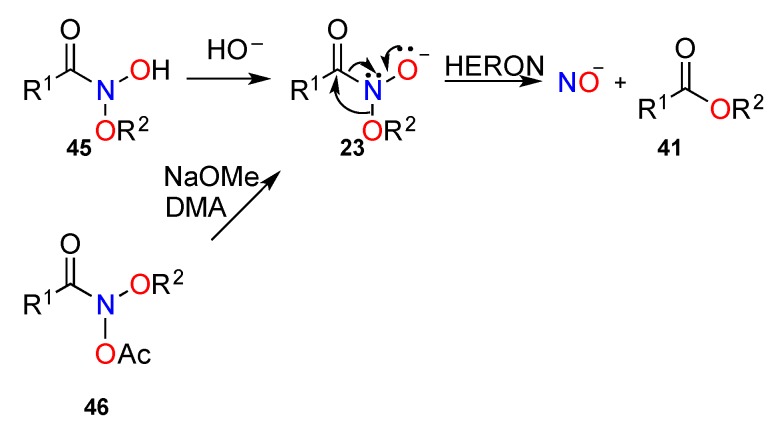
HERON reaction of hydroxamate anion **23**.

Hydroxide substitution of the acyloxyl side chain from a range of *N*-acyloxy-*N*-alkoxybenzamides **21**, at room temperature in aqueous acetonitrile, generated esters and their hydrolysis products [45]. Rate data and crossover experiments pointed to an S_N_2 reaction at nitrogen and the intramolecular nature of the reaction, implicating a HERON process. The hydroxamic acid intermediates **45** from initial attack (Scheme 1 iii) would be converted to their conjugate base **23** under basic reaction conditions, generating a strong n_O−_–σ*_NO_ anomeric destabilisation of the N–O bond (Scheme 10). A HERON migration of the alkoxyl side chain to the carbonyl carbon results in the formation of alkyl benzoate and the ejection of the nitric oxide anion (Scheme 10, R^1^ = Ph). This route to esters from hydroxamic acids in equilibrium with **23** was earlier invoked to explain non-crossover ester formation in the A_Al_1 solvolysis of *N*-acyloxy-*N*-alkoxyamides at low acid concentrations (Scheme 5) [45,46,48,49].

The HERON reaction was also invoked by Shtamburg and coworkers to account for formation of ethyl benzoate **41** (R^1^ = Ph, R^2^ = Et) when *N*-acetoxy-*N*-ethoxybenzamide **46** (R^1^ = Ph, R^2^ = Et) was treated with methoxide in aprotic media. Anion **23** (R^1^ = Ph, R^2^ = Et) leading to the HERON reaction was generated by methoxide addition at the ester carbonyl (Scheme 10), while methoxide attack at the amide carbonyl lead to the formation of methyl benzoate [122].

**Scheme 11:**
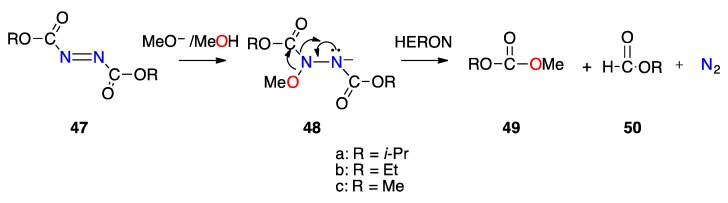
Amide anion induced HERON reaction in reactions of azadicarboxylates with methoxide.

Dialkyl azadicarboxylates, widely used in the Mitsonobu reaction, decompose vigorously with methoxide in methanol [123,124]. **47a** afforded methyl isopropyl carbonate **49a** and isopropyl formate **50a** in a 1:1 ratio by ^1^H NMR, and diethyl azadicarboxylate **47b** behaved similarly, though volatile ethyl formate **50b** was less prevalent in the reaction mixture (Scheme 11) [112,125]. Since the nitrogens in **47** are the overwhelming contributors to the LUMO of azadicarboxylates [112], the most probable route to these products is methoxide addition at nitrogen and a facile HERON reaction of the anionic adducts **48**. Calculations based on the HERON reaction of dimethyl azodicarboxylate **5c**, gave an *E*_A_ of 27 kJ mol^−1^ and exothermicity of 59 kJ mol^−1^, at the B3LYP/6-31G*//HF/6-31G(d) level [112].

#### 3.2.5. HERON Reactions of *N*-Alkoxy-*N*-Aminocarbamates

**Scheme 12:**
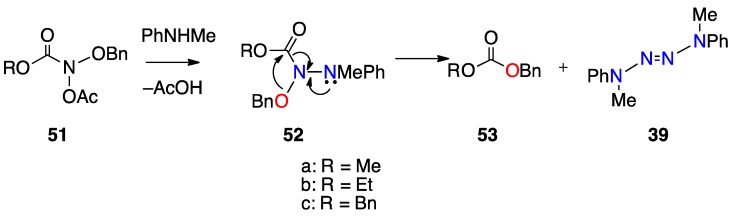
HERON reactions of *N*-alkoxy-*N*-aminocarbamates.

By analogy with the HERON reactions of *N*-acyloxy-*N*-alkoxyamides, several carbamates have been shown to undergo a similar reaction (Scheme 12). *N*-Acetoxy-*O*-alkyl-*N*-benzyloxycarbamates **51a**–**c** and *N*-methylaniline reacted bimolecularly in [D_4_]-methanol to produce the corresponding carbonates **53** and tetrazene **39**, presumably through HERON reaction of the *N*-methylanilino intermediate **52** [126].

#### 3.2.6. HERON Reactions of *N*-Acyloxy-*N*-Alkoxyamides

Based on RE’s of models **15c** and **16a** in Table 4, the amidicity of NNO systems such as *N*-alkoxy-*N*-aminoamides **2d** or *N*-alkoxy-*N*-aminocarbamates such as **52**, is likely to be reduced by modest amounts (60–70% that of *N*,*N*-dimethylacetamide), yet these systems undergo HERON reactions at room temperature, as do the 1,1-diazenes **2h** and hydroxamates **2g**. However, all have in common a strong anomeric destabilisation of the N–O bond through high energy electron pairs on the donor atom, n_Y_, and high electronegativity of oxygen. n_O_–σ*_NO_ systems such as *N*-acyloxy-*N*-alkoxyamides **2b** and *N*,*N*-dialkoxyamides **2c**, on the other hand, should have lower RE’s (amidicities approximately 50% that of *N*,*N*-dimethylacetamide), yet they are thermally stable at room temperature on account of a weaker n_O_–σ*_NO_ anomeric interaction. However, at elevated temperatures, *N*-acyloxy-*N*-alkoxyamides **2b** also undergo HERON reactivity [85].

**Scheme 13:**
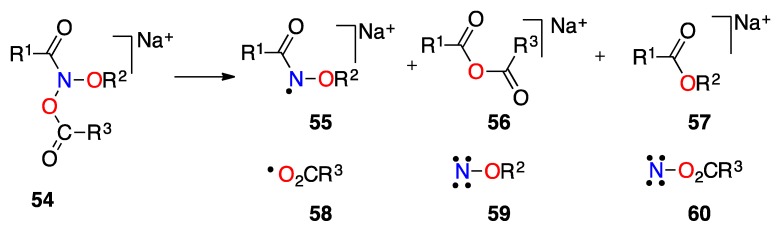
Fragments from collision induced electrospray ionisation-mass spectrometric (ES-MS) analysis of *N*-acyloxy-*N*-alkoxyamides **21**.

A tandem mass spectrometric analysis by electrospray ionisation, used in characterising mutagenic *N*-acyloxy-*N*-alkoxyamides **21** (Scheme 13), produced, in addition to sodiated parent compound **54**, three characteristic sodiated ions, a sodiated alkoxyamidyl radical, **55**, which was generally most prevalent, a sodiated anhydride, **56**, and a minor cation due to sodiated ester, **57**, which was absent in the ionisation of aliphatic amides. While the fragments formed in parallel with each product ion, **58**–**60**, were undetectable, under these conditions the source of anhydrides must be an intramolecular process and the HERON rearrangement at elevated temperature was implicated. Furthermore, the relative amounts of sodiated anhydride and ester reflected the expected bias towards an n_O_–σ*_NOAc_ anomeric stabilisation with attendant weakening of the N–OAc, rather than the N–OR, bond [112]. A B3LYP/6-31G(d) computational study of migration tendencies in *N*-formyloxy-*N*-methoxyformamide predicted high *E*_A_’s for migration of both formyloxyl and methoxyl in the gas phase (162 and 182 kJ mol^−1^, respectively) [112]. While acyloxyl, rather than alkoxyl, migration would be energetically more favourable, in solution at room temperature and particularly in polar media, the HERON reaction would not be competitive with S_N_2 and S_N_1 reactions at nitrogen.

**Scheme 14:**
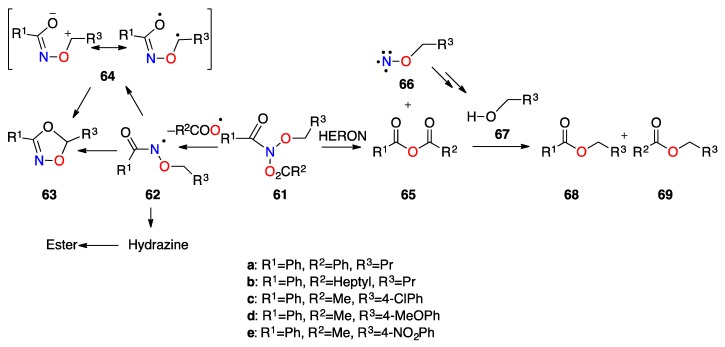
HERON and radical decomposition pathways for *N*-acyloxy-*N*-alkoxyamides in toluene at 90 °C.

**Figure 15:**
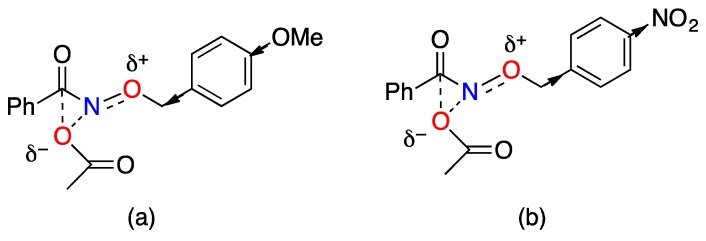
(**a**) Stabilisation of the HERON transition state for *N*-acyloxy-*N*-alkoxyamides by *para* methoxyl group and (**b**) destabilisation by a *para* nitro group.

However, in toluene at 90 °C, HERON reactions of **21** have been detected in competition with a homolytic decomposition pathway, and analysis of the complex reaction mixtures provides further support for the driving force behind the HERON process (Scheme 14) [85]. Homolysis of the N–OAc bond in **61** gave relatively long-lived alkoxyamidyl radicals **62**, which in solvent cage reactions with product radicals generated dioxazole **63**, or upon escaping the solvent cage dimerise to hydrazines leading to the expected thermal decomposition esters. The HERON reaction of **61** generates anhydrides **65** and alkoxynitrenes **66**. Anhydrides, which in the case of symmetrical benzoic anhydride from **61a** and mixed benzoyl heptanoyl anhydride from **61b** were relatively stable, react further to give esters **68** and **69** with alcohols **67** generated in the reaction mixture, the source of which was the alkoxynitrenes, the other HERON product. Critical evidence for the HERON process derived from products of alkoxynitrenes **66**, which (1) could be trapped by oxygen; (2) dimerised to hyponitrites; or, (3) underwent characteristic rearrangements leading aldehydes, nitriles, and alcohols. Competition between the HERON and homolytic reaction pathways was evident in a comparison of products from **61c**–**e**. The polarity of these HERON transition states would require a build-up of positive charge on the donor alkoxyl oxygen, n_O_. This would be stabilised by electron-donor *para* substituents on the benzyloxy group, but destabilised by electron-withdrawing *para* substituents. In accord with this, **61c** and **61e** generated dioxazole and esters. Little dioxazole was formed from **61d** in which the methoxyl group would lower the energy of the HERON transition state, but would have little impact on the non-polar transition state for homolysis of the N–OAc bond (Figure 15).

#### 3.2.7. HERON Reactions of *N*,*N*-Dialkoxyamides

Like *N*-acyloxy-*N*-alkoxyamides **2b**, *N*,*N*-dialkoxyamides **2c** possess low amidicity and they are thermally unstable, but require higher temperatures (typically 155 °C in mesitylene). However, their reaction proceeds exclusively by homolysis. Secondary products from alkoxynitrenes, which would be produced by the HERON pathway, were not observed for acyclic *N*,*N*-dialkoxyamides. Rather, they produce alkoxyamidyl radicals, which dimerise to *N*,*N*′-dialkoxy-*N*,*N*′-diacylhydrazines and ultimately esters. In addition, they can be trapped by hydrogen donors and solvent derived radicals [55].

**Scheme 15:**
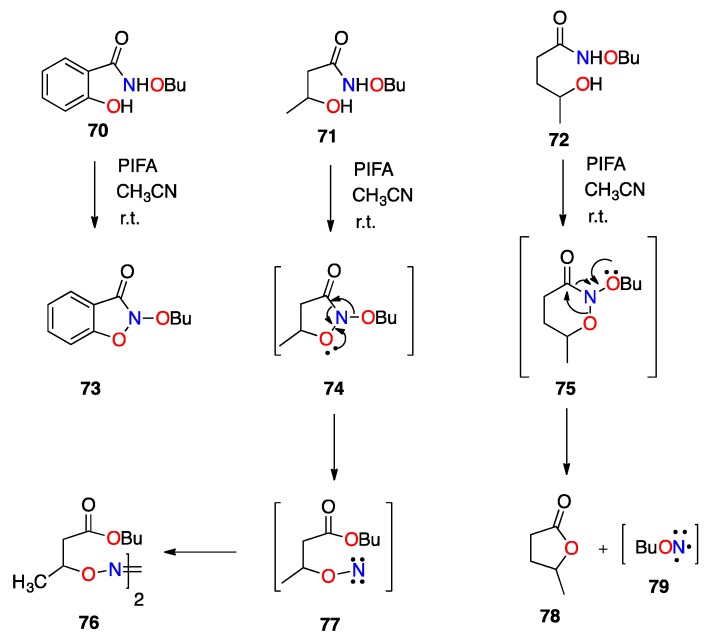
HERON reactions of γ-oxazinolactam **71** and δ-oxazinolactam **72**.

On the other hand, room temperature HERON reactivity was found to occur exclusively in several alicyclic ONO systems [30]. Cyclic *N*,*N*-dialkoxyamides, *N*-butoxy-3(*2H*)-benzisoxazolone **73**, *N*-butoxyisoxazolidin-3-one **74**, and *N*-butoxytetrahydro-*2H*-1,2-oxazin-3-one **75,** can be synthesised by PIFA oxidation of the salicamide **70**, β^−^ and γ-hydroxyhydroxamic esters, **71** and **72**, respectively, by analogy with the synthesis of acyclic *N*,*N*-dialkoxyamides (Scheme 4) [55]. Only *N*-butoxy-3(2*H*)-benzisoxazolone **73** is stable; *N*-butoxyisoxazolidin-3-one **74** and *N*-butoxytetrahydro-2*H*-1,2-oxazin-3-one **75** both react at room temperature and the reactions can be monitored by ^1^H NMR and mass spectrometry (Scheme 15). The γ-oxazinolactam undergoes quantitative ring opening to a diastereomeric mixture of the stable hyponitrite **76**, which must arise from dimerization of alkoxynitrene **77**, a known reaction of alkoxynitrenes. The δ-oxazinolactam, on the other hand, undergoes a quantitative ring contraction to the γ-valerolactone **78** with production of butoxynitrene **79**. Both are clearly HERON reactions [30].

**Figure 16:**
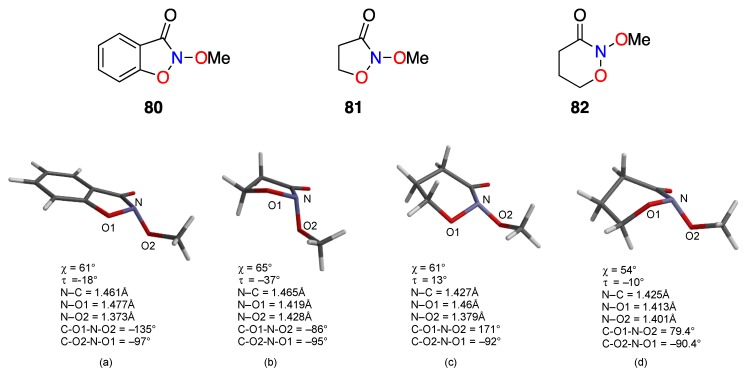
Ground state structures of models (**a**) *N*-methoxybenzisoxazolone **80**, (**b**) *N*-methoxy-γ-oxazinolactam **81**, and (**c**) chair and (**d**) boat *N*-methoxy-δ-oxazinolactam **82**.

The *E*_A_ for both HERON reactions must be radically lower than that for acyclic *N*,*N*-dialkoxyamides. Analysis of the B3LYP/6-31G(d) optimised ground state structures of *N*-methoxy-γ-oxazinolactam **81** (Figure 16b) and *N*-methoxy-δ-oxazinolactam **82** (Figure 16c) provide insight into this unusual difference in reactivity. Firstly, the model γ-oxazinolactam is strongly pyramidal at nitrogen (χ_N_ = 64.6°) and significantly twisted (τ = −36°), with attendant loss of amide character. The N–C(O) bond is very long compared to *N,N*-dimethoxyacetamide **4b** (1.417 Å, Table 3). The COSNAR and TA resonance energies for **81** are −20 and −19 kJ mol^−1^, respectively, translating to amidicities of only 26% and 25%. Torsion angles close to 90° indicate that the *endo* and *exo* oxygen lone pairs are ideally aligned for maximum n_O_–σ*_NO_ stabilisation. Low amidicity and a strong stereoelectronic effect would favour either reaction but, clearly, ring opening would be more favourable than ring contraction, which would give a highly strained β-lactone.

The B3LYP/6-31G(d) structure of the model δ-oxazinolactam **82** (χ_N_ = 61° and τ = 13°) matches the experimental (^1^H NMR) chair conformation of **75** which has a chiral nitrogen. It is also more pyramidal at nitrogen and slightly more twisted than the alicyclic *N*,*N*-dimethoxyacetamide **4b** (χ_N_ = 48° and τ = 9°, Figure 7c, Table 3), but its resonance and amidicity (RE**_COSNAR_** = −38 kJ mol^−1^, amidicity 49%, and RE**_TA_** = −37 kJ mol^−1^, amidicity 47%) is almost identical to the open chain form. However, only an n_O(*exo*)_–σ*_NO(*endo*)_ anomeric alignment is evident; with a torsion angle of 171°, the n_O(*endo*)_–σ*_NO(*exo*)_ interaction is completely switched off. The *endo* N–O bond is nearly 0.1 Å longer than the *exo* N–O bond (the *endo* bond is marginally shorter than the *exo* bond by 0.01 Å in γ-oxazinolactam and N–O bonds differ by 0.02 Å in *N*,*N*-dimethoxyacetamide). Ring opening and ring contraction are computed to have about the same *E*_A_ and Δ*H*^‡^ at B3LYP/6-31G(d) [30]. It is evident that the ring contraction of the δ-oxazinolactam to γ-butyrolactone is largely driven by a strong, conformationally imposed anomeric effect, a remarkable impact of anomeric substitution at an amide nitrogen [30]. While the computed transition state for ring opening of model *N*-methoxy-δ-oxazinolactam is marginally lower in energy, the required n_O(*endo*)_–σ*_NO(*exo*)_ is only accessible from the boat conformation of the δ-oxazinolactam (Figure 16d). Experimentally, accessing this conformation in **75** by nitrogen inversion could be energetically unfavourable, owing to steric hindrance between the axial 4-methyl and *N*-butoxy groups. However, even in the boat conformation, the strong n_O(*exo)*_–σ*_NO(*endo*)_, which favours ring contraction is still evident.

The γ-lactam in benzisoxazolone is stable at room temperature, though model **80** has a moderately suitable anomeric alignment for migration of O2. However, the n_O(*endo*)_–σ*_NO(*exo*)_ interaction is probably weakened by conjugation of the O*_endo_* p-type lone pair onto the aromatic ring. Ring contraction driven by a favourable n_O(*exo)*_–σ*_NO(*endo*)_ interaction would be disfavoured.

#### 3.2.8. *N*-Alkoxy-*N*-Alkylthiylamides

**Scheme 16:**
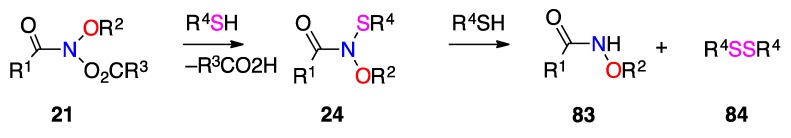
Reaction of *N*-acyloxy-*N*-alkoxyamides with alkylthiols.

The combination of sulphur and oxygen attachment to amide nitrogen in *N*-methoxy-*N*-methylthiylacetamide **15d** results in a similar reduction in amide resonance to that of *N*-methoxy-*N*-dimethylaminoacetamide **15c**, namely about 64% vs. 67% (Table 4). However, the reaction of *N*-acyloxy-*N*-alkoxyamides **21** with biological thiols, glutathione, and methyl and ethyl esters of cysteine, which resulted in S_N_2 displacement of carboxylate, produced exclusively hydroxamic esters **83** and disulphides **84** [52] (Scheme 16). The driving force for HERON reactivity is not only reduced resonance, but the anomeric effect. Instead of HERON reactions, the intermediate *N*-alkoxy-*N*-alkylthiylamides **24** undergo an S_N_2 reaction at sulphur by thiol. The distinction between reactivity modes for NNO and SNO systems lies in the n_S_–σ*_NO_ anomeric interaction, which is much weaker than the n_N_–σ*_NO_ of *N*-alkoxy-*N*-aminoamides.

### 3.3. Driving Force for the HERON Reaction

**Figure 17:**
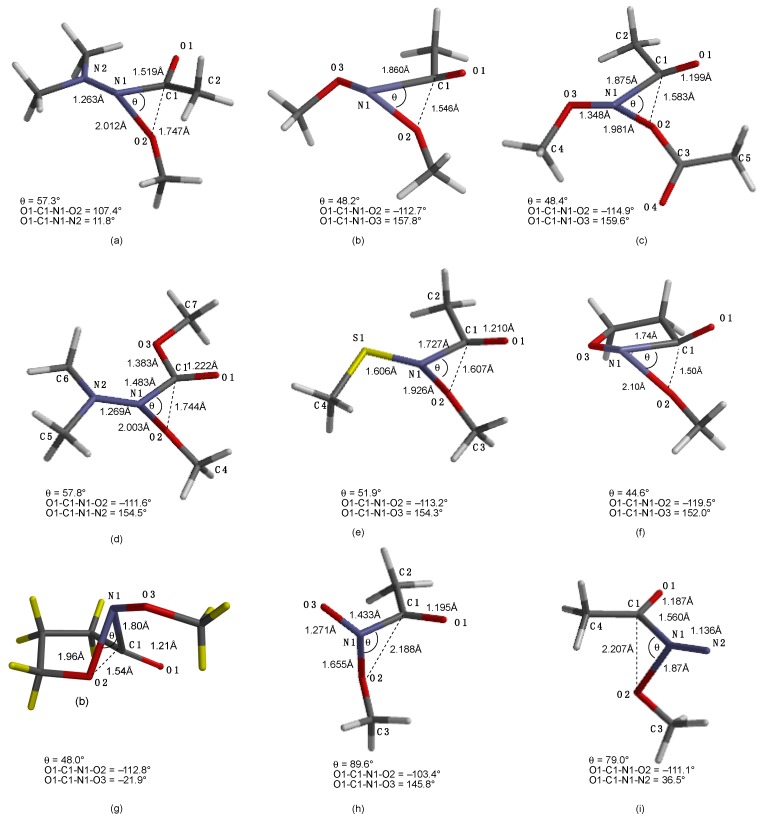
B3LYP/6-31G(d) optimised transition states of favoured HERON reactions of (**a**) *N*-methoxy-*N*-dimethylaminoacetamide **15c**, (**b**) *N*,*N*-dimethoxyacetamide **4b**, (**c**) *N*-acetoxy-*N*-methoxyamide **15b**, (**d**) *O*-methyl-*N*-methoxy-*N*-dimethylaminocarbamate **16a**, (**e**) *N*-methoxy-*N*-methylthiylacetamide **15d**, (**f**) *N*-methoxy-γ-oxazinolactam **81** (ring opening), (**g**) *N*-methoxy-δ-oxazinolactam **82** (ring contraction), (**h**) *N*-methoxyacetohydroxamate **15f** and (**i**) 1-acetyl-1-methoxydiazene **15g**.

The most accessible transition states for HERON migration of methoxyl in a number of model anomeric amides, *N*-methoxy-*N*-dimethylaminoacetamide **15c**, *N*,*N*-dimethoxyacetamide **4b**, *N*-acetoxy-*N*-methoxyacetamide **15b**, and *O*-methyl-*N*-methoxy-*N*-dimethylaminocarbamate **16a**, ring opening of *N*-methoxy-γ-oxazinolactam **81**, and ring contracting of *N*-methoxy-δ-oxazinolactam **82**, each of which represents a class of neutral anomeric amides known to undergo HERON reactions, as well as for methoxyl migration in *N*-methoxy-*N*-methylthiylacetamide **15d**, *N*-methoxyacetohydroxamate **15f**, and 1-acetyl-1-methoxydiazene **15g**, have been derived at the B3LYP/6-31G(d) level as part of several studies [30,61,62]. Transition state geometries of **4b**,**15b**–**d**,**f** and **g**, **16a**, **81**, and **82** are presented in Figure 17.

In all the transition state complexes, the migrating oxygen does so in a plane largely orthogonal to the N1–C1–O1 plane and the donor atom n_Y_, driving the migration, is largely in the N1–C1–O1 plane. As a consequence, the amide nitrogen lone pair lies close to the plane and amide resonance is largely lost in the transition state. The resonance energy, RE is therefore a component of the overall *E*_A_, the balance being the energy required for the rearrangment under anomeric assistance, *E*_rearr_, and must reflect the relative nature of the n_X_–σ*_NO_ driving force. It is therefore possible to approximate the influence of the anomeric substituents on the resonance interaction on the one hand, and on the migration process, on the other. Table 8 gives the *E*_A_, RE_TA_, and net *E*_rearr_ data for these transition states.

Methoxyl migration in the NNO systems **15c** and **16a** (*E*_A_’s 95 and 92 kJ mol^−1^ respectively) have similar RE’s of around −50 kJ mol^−1^, and therefore *E*_rearr_ of approximately 40 kJ mol^−1^. The change in charge on the carbonyl carbon in the HERON transition state is negligible [76], so replacement of methyl by methoxyl has little bearing on the rearragement energies. ONO in **4b** and ONOAc in **15b** migrations have much higher *E*_A_’s despite the lower amide resonance energy. The large difference lies in the *E*_rearr_ which is nearly 100 kJ mol^−1^ less favourable and a reflection of the relative efficacy of the n_N_–σ*_NO_ vs. the weaker n_O_–σ*_NO_ anomeric interaction. The difference of about 80 kJ mol^−1^ between methoxy migration in the ONS **15d** and ONN acetamides **15c** lies, again, in the much weaker n –σ*_NO_ anomeric effect. This can be accounted for by size mismatch due the larger 3p orbitals of the sulphur. Both HERON reactions of the cyclic forms of *N*,*N*-dialkoxyamides **81** and **82** have substantially lower overall *E*_A_’s than *N*,*N*-dimethoxyacetamide **4b**. After RE has been taken into account, *E*_rearr_ values indicate that in the cyclic forms, reorganisation to the HERON transition state is easier. Better stereoelectronic control in the cyclic system accounts for this.

The RE of the 1,1-diazene **15g**, is computed to be about the same as the overall *E*_A_ for its HERON reaction. This is a very early transition state in keeping with the exothermicity of the process. *E*_rearr_ is essentially zero on account of the high energy electron-pair on the amino nitrene and the *E*_A_ is essentially equivalent to the RE that must be sacrificed. Likewise, the hydroxamate **15f** bears a very high energy electron pair on the anomeric donor oxygen. While the resonance energy is similar to that of *N*,*N*-dimethoxyacetamide, the *E*_rearr_ is small. This too is a very early transition state.

Overall, the decreasing order of *E*_rearr_ of XNY systems based on the deconvolution in Table 8, which can be regarded as the order of decreasing effectiveness of an n_Y_–σ*_NX_ interaction and destabilisation of the N–OMe bond, is AcONO > ONS ~ ONO > ONN > ONO^−^ > ONNitrene. The order of RE**_TA_** is ONN > ONS > AcONO > ONO > ONO^−^ > ONNitrene and the overall activation energies decrease in the order AcONO > ONS > ONO > ONN > ONO^−^ > ONNitrene. Clearly, the dominant influence in HERON reactivity is the strength of the anomeric effect rather than the decrease in amidicity.

**Scheme 17:**
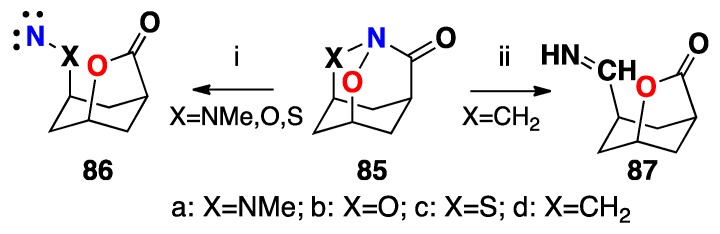
HERON migrations of alkoxyl in the absence of resonance.

**Scheme 18:**
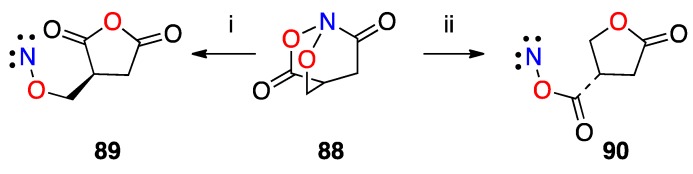
HERON reactions of *N*-acyloxy-*N*-alkoxyamide in absence of resonance.

Rearrangement energies, *E*_rearr_’s, obtained by the deconvolution method represent activation energies in the absence of resonance. Relative *E*_rearr_’s can be compared to relative *E*_A_’s for intramolecular rearrangement in fully twisted amides, heteroatom-substituted 1-aza-2-adamantanones **85** (Scheme 17) and 2-quinuclidone **88** (Scheme 18) and the *E*_A_’s are also presented in Table 8. Δ*E*_A_’s relative to migration of oxygen (Scheme 17i) in **85b**, for **85a**, and **85c** are −88 and 5.8 kJ mol^−1^, respectively, which correlate well with the respective differences in *E*_rearr_ of the respective acetamides, namely −77 and 5 kJ mol^−1^. Similarly, the difference in the ease of migration of alkoxyl to form **90** and acyloxyl to form **89** in **88** (Scheme 18i,ii) is 28 kJ mol^−1^ and the difference between the corresponding *E*_rearr_ form the *syn* conformer of **15b** is 27 kJ mol^−1^.

No transition state can be found for migration of alkoxyl group in hydroxamic ester **3b**. However, **85d** can be rearranged to lactone **87** (Scheme 17ii) with concomitant rearrangement of the nitrene product to the imine. The difference between *E*_A_ for this process and the rearrangement of **85a** is 184 kJ mol^−1^. If this translates to the difference in *E*_rearr_ between twisted *N*-methoxy-*N*-methylacetamide **3b** and *N*-methoxy*-N*-dimethylaminoacetamide **15c**, for which *E*_rearr_ is 43.5 kJ mol^−1^, the rearrangement of **3** to methyl acetate and methylnitrene would have an activation energy of about 233 kJ mol^−1^ from twisted **3b** or, adding in the RE for **3b** of 62 kJ mol^−1^, 295 kJ mol^−1^ from conjugated **3**. It is clear that hydroxamic esters do not rearrange to esters and alkylnitrenes and the role of anomeric (bisheteroatom) substitution in the HERON reaction is vital.

## 4. Conclusions

In this review we have outlined the theoretical and structural properties and the reactivity of the class of anomeric amides. Much of the data from this, and several other groups, is relatively recent and, while several compilations on the subject have appeared in the literature, a focus on the perturbation of the amide structure is befitting this special edition. The fairly recent accrual of structural data from our laboratory and that of Shtamburg has provided experimental verification of the unusual properties of bisheteroatom-substituted amides. Coupled with extensive computational results, the effect of heteroatoms at nitrogen on amide resonance and conformation at the amide nitrogen can be better understood and predicted, in particular the role played by electronegativity of the bonded atoms and orbital interactions. Ensuing from the work is a clearer understanding of the energetic consequences of distortion of the amide linkage. It is clear that spectroscopic properties of various congeners are dictated by two principle concepts: the first is impaired resonance owing to change in hybridisation bought about by orbital interactions that have their foundation in Bent’s, and more recently Alabugin’s theories of orbital interactions. Of particular importance is the reassignment of 2s character to the amide nitrogen lone pair orbital, as consequence of both electronegativity and orbital overlap considerations. Secondly, the participating atoms in this purturbation of regular amide bonding possess intrinsic orbital interactions by virtue of both their electronegativity and their lone pairs. The anomeric interaction bought about by n_Y_–σ*_NX_ overlap is pronounced in anomeric amides. What is clear is that the electronegativity induces a shift to less resonance through pyramidalisation and lowering the energy of the amide nitrogen lone pair and the anomeric interaction is better served by this shift to sp^3^ character. Stronger electronegativity serves to reduce resonance as well as to promote the anomeric interaction.

Regarding reactivity, it is abundantly clear that both resonance impairment and strength of the anomeric effect are definitive, but the anomeric effect dominates in promoting both S_N_2 and elimination reactions at the amide nitrogen. Most significant is the anomeric driving force for the HERON reaction, notably in systems such as *N*-amino-*N*-alkoxyamides (NNO systems) where the energetics for anomeric weakening of the N–O bond are optimised, and in cyclic systems such as the *N*-alkoxy-γ- and δ-oxazinolactams where conformation and configuration appear to favour and enhance anomeric overlap. The HERON reaction is unique to this class of amides and has no equivalence in the literature. It operates in the opposite sense to the well-known Curtius, Hoffman, and Lossen rearrangements in which an acyl substituent migrates from the carbonyl to the nitrogen. Finally our studies of the HERON and those of Barton and coworkers, shed light on the well-known decomposition reactions of *N*,*N*′-dialkoxy-*N*,*N*′-diacylhydrazines. The HERON mechanism that operates in these decompositions and in those of the 1-acyl-1-alkoxydizenes is critical to the synthesis of highly hindered esters.

## Figures and Tables

**Table 1:** Selected structural data for anomeric amides **5**–**9** from X-ray structures (Figure 6).

Structure	N–C(O)/Å	(N)C=O/Å	θ/°	χ_N_/°	τ/°	Anomeric Twist ^1^*/°*
**5a** [33]	1.409	1.206	331.2	58.3	6.7	**C10-O3-N1-O2: 95.9** C8-O2-N1-O3: −114.1
**5b** [33]	1.421	1.211	334.5	55.6	13.9	**C8-O2-N1-O3: −101.6** C15-O3-N1-O2: −63.8
**6a** [29]	1.439	1.205	323.5	65.3	15.5	**C18-O3-N1-O1:96.2** C7-O1-N1-O3: −141.6
**6b** [29]	1.441	1.207	324.1	65.6	13.9	**C14-O3-N1-O1:96.7** C7-O1-N1-O3: −137.6
**7** [65]	1.411	1.203	330.3	59.7	−14.0	**C8-O1-N1-O5:−91.8** C9-O5-N1-O1: 116.2
**8**(N1) [32]	1.412	1.213	343.2	47.3	−8.5	LP (N2)-N2 -N1-O2: 47.3
**8**(N2)	1.410	1.207	341.1	48.9	−11.4	**LP (N1)-N1-N2-O3: 178.6**
**9** [65]	1.408	1.204	337.5	52.5	−13.3	**C8-O2-N1-Cl1: −84.2**

^1^ Anomeric alignments in bold-face.

**Table 2:** Selected structural data for ureas **10**–**12** and carbamates **13**–**14** from X-ray structures.

Structure	N–C(O)/Å	(N)C=O/Å	θ/°	χ_N_/°	τ/°	Anomeric Twist ^2^*/°*
**10a** [66]	1.443 ^1^	1.226	329.1	59.9	8.2	**C-O-*N*-Cl: –90.9**
**10b** [66]	1.472 ^1^	1.210	325.8	61.9	−13.4	**C-O-*N*-Cl: –100.1**
**11a** [67]	1.426 ^1^	1.222	333.6	−57.1	−6.8	**C-O-*N*-Oacyl: –104.0**
**11b** [68]	1.441 ^1^	1.233	323.7	64.6	11.8	**C-O-*N*-Oacyl: –98.2**
**12** [68]	1.438 ^1^	1.220	331.8	57.4	0.8	**C-O2-N1-O1:–89.3** C-O1-N1-O2: 55.2
**13** [67]	1.424	1.198	334.2	−56.3	2.9	**C-O-*N*-Oacyl: –95.5**
**14** [69] (N1)/(N2) ^3^	1.408	1.194	340.0	59.8	−9.4	**LP (N1)-N1-N2-O: 189.2**

^1^ Anomeric amide bond; ^2^ Anomeric alignments in bold-face; ^3^ Equivalent nitrogens.

**Table 3:** Selected structural data for B3LYP/6-31G(d) lowest energy conformers of model acetamides **4b**, **15a**–**e**, and **16a**,**b**.

Structure	N–C(O)/Å	(N)C=O/Å	(N–X,*N*–Y)/Å	θ/°	χ_N_/°	τ/°	Anomeric Twist Angles ^1^*/°*
**15a** ONCl [34]	1.432	1.207	Cl:1.787 O2:1.389	337.6	52.3	−5.3	**C2-O2-N1-Cl: 88.8**
**15b** ONOAc [61]	1.429	1.209	O2:1.423 O3:1.395	332.1	58.0	2.3	**C4-O3-N1-O2: 101.1**
**4b** ONO [33]	1.417	1.212	O2:1.387 O3:1.412	342.9	48.1	8.5	C2-O2-N1-O3: 66.6 **C3-O3-N1-O2: 83.8**
**15c** NNO [62]	1.404	1.217	O1:1.430 N2:1.387	346.5	41.8	5.4	**LP (N2)-N2-N1-O1: 190**
**15d** ONS [61]	1.408	1.215	S1:1.717 O2:1.420	352.4	31.7	−4.6	C4-S1-N1-O2: −79 **C3-O2-N1-S1: −86.6**
**15e** NNCl [34]	1.414	1.209	Cl:1.820 N2:1.351	360.0	0	0	**LP(N2)N2-N1-Cl: 180**
**16a** NNO [61]	1.406	1.212	O1:1.404 N2:1.383	342	46.3	0	**LP (N2)-N2-N1-O1: 169.4**
**16b** NNO	1.428	1.217	O1:1.397 N2:1.428	340.0	49.6	−1.7	**LP (N2)-N2-N1-O1: 167.5**

^1^ Anomeric alignment in bold face.

**Table 4:** B3LYP/6-31G(d), B3LYP/6-311++G(d,p), and M06/6-311++G(d,p) derived resonance energies and amidicities of model anomeric acetamides **4b**, **15a**–**e**, and **16a**.

Amide (R = Me)	Δ*E*_COSNAR_^1^ /kJ mol^−1^	Δ*E*_TA_ /kJ mol^−1^	Δ*E*_ind_ /kJ mol^−1^	RE_TA_^1, 2^ /kJ mol^−1^
**15a** (ONCl) [34]	−29.6(38)	69.9	23.4	−29.5(39)
**15a** ^3^	−27.2 (36)			
**15a** ^4^	−34.4(45)			
**15b** (ONOAc) [61]	−39.7(52)	65.3	29.7	−40.5(53)
**15b** ^4^	−39.5 (52)			
**4b** (ONO) [35]	−36.0(47)	58.2	18.0	−36.0(47)
**4b** ^4^	−39.5(53)		-	
**15c** (ONN) [62]	−52.3(69)	35.1	10.0	−51.0(67)
**15c** ^4^	−55.7(73)			
**15d** (ONS) [61]	−48.6(64)	26.8	5.0	−48.6(64)
**15d** ^4^	−47.3(62)			
**15e** (NNCl) [34]	−28.7(38)	64.5	17.6	−29.0(37)
**15e** ^3^	−28.6(38)			
**15e** ^4^	−48.7(64)			
**16a** (ONN) [61]	−47.7(63)	31.8	14.6	−50.6(67)
**1**	−75.9(100)			
**1**^4^ [34]	−75.9(100)			

^1^ Amidicity (%) in parentheses; ^2^ From Equation (4); ^3^ At B3LYP/6-311++G(d,p);^4^ M06/6-311++G(d,p).

**Table 5:** Typical spectroscopic data for stable anomeric alkanamides and arylamides (RC(O)NXY), and their hydroxamic ester precursors.

System	X	Y	R	Amide ν/cm^−1^ (δ^13^C)	Hydroxamic Ester ν/cm^−1^ (δ^13^C)
**2a** [79]	Cl	OBu	Me	1740 ^1^(175.3)	1678 (167.9)
**2a** [35]	Cl	OBu	Ph	1719 (174.2)	1654 (165.7)
**2b** [79]	OAc	OBu	Me	1746 (176.2)	1678 (167.9)
**2b** [80]	OAc	OBu	Ph	1732 (173.9)	1654 (165.7)
**2c** [81]	OBu	OBu	Me	1707 (174.1)	1678^1^ (167.9)
**2c** [55]	OMe	OMe	Ph	1711 (174.3)	1683 (166.4)
**2d** [76]	4-MeBnONAc	4-MeBnO	Me	1734/1700 (171.3)	1693 (168.0)
**2d** [76]	BzNOEt	OEt	Ph	1708 (170.0)	1685 (166.5)

^1^ Neat.

**Table 6:** Hammett reaction constants, Arrhenius activation energies, entropies of activation, and rate constants for HERON decomposition of **19a**–**e** and **20a**–**e** [76].

Series	System	*E*_A_/kJ mol^−1^	Δ*S^‡^*/J K^−1^ mol^−1^	10^6.^*k*_298_/s^−1^
Series **19**: σ^+^ reaction constant ρ = −0.35 ^1^	**19a**	99.2 (4.3)	−21.2 (13)	5.56
	**19b**	100.7 (8.2)	−19.6 (25)	3.72
	**19c**	100.4 (5.1)	−23.8 (15)	2.52
	**19d**	107.7 (1.7)	0.9 (5)	2.50
	**19e**	104.0 (1.7)	−15.4 (5)	1.57
Series **20**: σ reaction constant ρ = +1.02 ^1^				**10^8.^*k*_298_/s^−1^**
	**20a**	111.4 (0.9)	−21.1 (2)	4.02
	**20b**	125.9 (1.0)	24.5 (3)	2.76
	**20c**	114.1 (3.9)	−8.7 (11)	6.21
	**20d**	125.1 (8.5)	27.4 (24)	5.44
	**20e**	98.8 (0.3)	−46.4 (1)	31.9

^1^ Hammett correlations using *k*_298K_.

**Table 7:** Ester **41** (R^1^COOR^2^) formation from the reaction of *N*-alkoxy-*N*-chloroamides **25** (R^1^CONClOR^2^) with sodium azide in aqueous acetonitrile.

R	R′	Isolated Crude Yield/%
Ph	(CH_3_)_3_C	87
(CH_3_)_3_C	(CH_3_)_3_C	30
1-adamantyl	(CH_3_)_3_C	82
(CH_3_)_3_C	cyclohexyl	97
Ph	(CH_3_)_2_CH	92
Ph	PhCH_2_	93
CH_3_	PhCH_2_	92
p-NO_2_C_6_H_4_	Et	94
Ph	Et	94

**Table 8:** B3LYP/6-31G(d) activation energies (*E*_A_), resonance energies (RE_TA_), and rearrangement energies (*E*_rearr_) for HERON reactions of model anomeric amides and tricyclic anomeric amides; all values in kJ mol^−1^^.^

Migratory Mode	*E*_A_	XNY ^1^	RE_TA_^2^	*E*_rearr_
MeO from *anti* **15c**	95.0	ONN	−51.5	43.5
MeO from **4b**	156.5	ONO	−35.9	120.5
MeO from *syn* **16a**	92.4	ONN	−50.7	41.8
AcO from *syn* **15b**	181.0	AcONO	−40.5	140.5
MeO from *syn* **15b**	207.9	ONOAc	−40.5	167.4
AcO from *anti* **15b**	181.5	AcONO	−40.5	141.0
MeO from *anti* **15b**	182.8	ONOAc	−40.5	142.3
MeO from *anti* **15d**	174.1	ONS	−48.6	125.4
MeO from **15f** ^3^	48.0	ONO^–^	−32.4	15.6
MeO from **15g**	8.8	ONNitrene	−14.8	−6.0
Ring opening **81**	113.0	ONO	−19.2	93.8
Ring contraction **82**	145.2	ONO	−36.7	108.4
Ring opening of **82**	136.4	ONO	−36.7	99.7
Scheme 17 i X=NMe	59.0	ONN	0.0	59.0
Scheme 17 i X=O	146.9	ONO	0.0	146.9
Scheme 17 i X=S	152.7	ONS	0.0	152.7
Scheme 17 ii X=CH_2_	242.7	ONCH_2_	0.0	242.7
Scheme 18 i	133.5	AcONO	0.0	133.5
Scheme 18 ii	161.5	ONOAc	0.0	161.5

^1^ n_Y_ donor to σ*_NX_; ^2^ Transamidation data (COSNAR data very similar); ^3^ HF/6-31G(d) values.
